# Gene-based calibration of high-throughput functional assays for clinical variant classification

**DOI:** 10.1101/2025.04.29.651326

**Published:** 2025-05-04

**Authors:** Daniel Zeiberg, Malvika Tejura, Abbye E. McEwen, Shawn Fayer, Vikas Pejaver, Alan F. Rubin, Lea M. Starita, Douglas M. Fowler, Anne O’Donnell-Luria, Predrag Radivojac

**Affiliations:** 1 Khoury College of Computer Sciences, Northeastern University, Boston, MA 02115, USA;; 2 Department of Genome Sciences, University of Washington, Seattle, WA 98195, USA;; 3 Brotman Baty Institute for Precision Medicine, University of Washington, Seattle, WA 98195, USA;; 4 Department of Laboratory Medicine and Pathology, University of Washington, Seattle, WA 98195, USA;; 5 Institute for Genomic Health and Department of Genetics and Genomic Sciences, Icahn School of Medicine at Mount Sinai, New York, NY 10029, USA;; 6 Bioinformatics Division, The Walter and Eliza Hall Institute of Medical Research, Parkville, VIC, Australia; Department of Medical Biology, University of Melbourne, Melbourne, VIC, Australia;; 7 Department of Bioengineering, University of Washington, Seattle, WA 98195, USA;; 8 Program in Medical and Population Genetics, Broad Institute of MIT and Harvard, Cambridge, MA02142, USA;; 9 Division of Genetics and Genomics, Boston Children’s Hospital, Harvard Medical School, Boston, MA 02115, USA.

## Abstract

High-throughput functional assays measure the effects of variants on macromolecular function and can aid in reclassifying the rapidly growing number of variants of uncertain significance. Under the current clinical variant classification guidelines, using functional data as a line of evidence to assert pathogenicity relies on determining assay score thresholds that define variants as functionally normal or functionally abnormal. These thresholds are designed to maximize the separation of variants with known clinical effects (benign, pathogenic) and often incorporate expert opinion. However, this approach lacks the rigor of calibration, in which a variant’s posterior probability of pathogenicity must be estimated from the raw experimental score and mapped to discrete evidence strengths. To build upon the existing guidelines, we introduce and evaluate a method for calibrating continuous high-throughput functional data as a line of evidence in clinical variant classification. Assay score distributions of synonymous variants and variants appearing in gnomAD for a given functional scoreset are jointly modeled with score distributions of known pathogenic and benign variants using a multi-sample skew normal mixture of distributions. This model is learned using a constrained expectation-maximization algorithm that provably preserves the monotonicity of pathogenicity posteriors and is subsequently used to calculate variant-specific evidence strengths for use in the clinic. Using 24 datasets from 14 genes, we first assess the model’s ability to capture assay score distributions. We then demonstrate its potential impact on reclassifying variants by comparing the evidence strengths assigned at the variant-level with those assigned uniformly to all functionally normal and abnormal variants under the existing ClinGen guidelines. An improved classification of variants will directly improve the accuracy of genetic diagnosis and subsequent medical management for individuals affected by Mendelian disorders.

**Availability:**

https://github.com/dzeiberg/mave_calibration

## 1 Introduction

Genetic diagnosis is the cornerstone of genomic medicine for rare disease [46, 49]. It aims to identify one or a handful of causal variants in a patient’s genome based on the wealth of sequence, functional, and clinical data. To standardize the approach, the American College of Medical Genetics and Genomics (ACMG) and the Association for Molecular Pathology (AMP) maintain evidence-based guidelines for classifying variants in genes causally related to Mendelian disease [45]. A variant reported to a clinician can be classified into one of the five categories: “pathogenic”, indicating a 99% probability it causes a Mendelian disease; “likely pathogenic”, indicating a 90% probability it causes disease; “likely benign”, indicating a 90% probability it does not cause disease; “benign”, indicating a 99% probability it does not cause disease; and “variant of uncertain significance” (VUS), covering the intermediate range of probabilities. A variant’s classification is conditioned on lines of available evidence supporting or refuting its disease causality. The 2015 guidelines designate the type (e.g., functional, computational, population, or segregation data), strength (supporting, moderate, strong, very strong, standalone), and direction (benign, pathogenic) of each line of evidence, and include a set of rules for combining evidence to classify a variant into one of the five categories [45]. To be classified as likely pathogenic or pathogenic, variants must have at least two lines of evidence of sufficient strength (e.g., two lines of strong evidence for likely pathogenic) that combine to support their clinical classification. These variants are considered diagnostic and likely to be clinically actionable [44].

The ACMG/AMP system has been approximated by a single-parameter Naïve Bayes classifier in which the strength of evidence is quantified using its positive likelihood ratio (${\text{LR}}^{+}$), also referred to as OddsPath, where discrete evidence strengths (supporting, moderate, etc.) have exponentially increasing ${\text{LR}}^{+}$ [51]. However, implementing this probabilistic framework necessitates methodology for calibrating individual lines of evidence, which in turn requires estimation of ${\text{LR}}^{+}$ as well as the prior probability of pathogenicity in a reference variant set. For each evidence source, calibration aims to map a concrete score (e.g., computational prediction, case-control counts, functional assay readout) to discrete evidence of a specific direction and strength to support the ACMG/AMP rule-based evidence combination.

Pejaver et al. [39] proposed a nonparametric method for calibrating computational predictors for missense variation by estimating a rare variant’s prior probability of pathogenicity in disease-associated genes as well as local positive likelihood ratios (${\text{lr}}^{+}$) of real-valued scores using a validated set of pathogenic and benign variants from ClinVar [26]. They demonstrated that several models exceed the supporting evidence strength assigned to computational predictors in the initial guidelines, resulting in updated ClinGen recommendations that allow these predictors to reach evidence levels up to strong [39, 4]. Subsequent studies have shown that the calibration works as intended in a genetic diagnosis setting [48], although the performance levels vary across genes [53].

Despite the increasing discriminative power of computational tools over time [54, 43], predictors alone are not sufficient to classify a rare variant as pathogenic and, thus, additional lines of evidence are needed [45]. Functional assays, including multiplexed assays of variant effect, are being deployed [20] to generate the evidence needed to reclassify the rapidly increasing number of VUS and prospectively quantify the effect of variants yet to be observed in the clinic [50, 15]. To standardize the use of functional evidence across clinical laboratories, the ClinGen [44] Sequence Variant Interpretation (SVI) Working Group established recommendations for using functional data in clinical variant classification [6]. The recommendations included steps for determining the validity and strength of functional evidence that can be applied to an individual variant. All variants with assay scores exceeding the specified thresholds for functionally normal or abnormal are assigned a fixed evidence strength, determined by expert opinion or by the level of separation of known pathogenic and benign variants. Such threshold determination is a potential source of inconsistency in the use of functional evidence. Additionally, the grouping of all variants exceeding thresholds may over- or under-estimate evidence strength for variants near and far from the thresholds.

A distinction between calibrating computational predictors and high-throughput functional assays is that, unlike predictor scores, assay scores cannot readily be compared across individual studies, as each assay score quantifies a particular function of a variant relative to the function of the wild type. The often limited number of clinical control variants in each gene poses additional challenges, making it difficult to repurpose the previously proposed method for predictor calibration. Finally, in addition to assaying a large number of missense variants, a modern high-throughput functional experiment usually includes a set of assayed synonymous variants. The scores of these synonymous variants can be exploited to model the score distribution of functionally neutral variation as well as the experimental variability of assay scores.

To increase consistency in the use of functional evidence across clinical laboratories, we here propose a statistical method for calibrating high-throughput functional data. We focus on a curated set of 24 functional datasets that quantify the functional effect of missense variants in 14 genes. We demonstrate that this methodology determines assay score thresholds that define variant-level evidence strength for these datasets and highlight the method’s potential for reclassifying variants.

## 2 Materials and Methods

The objective in calibrating high-throughput functional data for a given gene or domain is to quantify the strength of evidence an assay score s for a variant v can serve in classifying v as pathogenic or benign. That is, we seek to learn a mapping function f:R→𝒮×𝒟 from an assay score s∈R into one of the discrete evidence strengths $𝒮=\left\{\text{supporting},\;\text{moderate},\;\text{strong},\;\text{very}\;\text{strong}\right\}$ and directions $𝒟=\left\{\text{benign},\;\text{pathogenic}\right\}$. Variants whose scores do not map into any of the categories above are said to have “indeterminate” strength and direction [39].

### 2.1 Calibration framework

To learn the calibration function, we will follow the general probabilistic framework based on the relationship between prior and posterior odds of pathogenicity [17] as

posterioroddsofpathogenicity=positivelikelihoodratio×prioroddsofpathogenicity.

Letting E be a discrete random variable representing evidence and Y be a binary random variable representing whether (Y=1) or not (Y=0) a variant causes a rare disease, the posterior odds of pathogenicity can be expressed as P(Y=1∣E=e)P(Y=0∣E=e), the prior odds of pathogenicity as P(Y=1)P(Y=0), and the positive likelihood ratio ${\text{LR}}^{+}(e)$ as P(E=e∣Y=1)P(E=e∣Y=0). In the case evidence is produced by a single binary predictor, ${\text{LR}}^{+}$ is equivalent to the ratio of true positive rate and false positive rate.

To model the expert-derived rules from the ACMG/AMP guidelines [45], Tavtigian et al. [51] proposed to express ${\text{LR}}^{+}$ in an exponential form as

(1) $${\text{LR}}^{+}=c^{\frac{n_{\text{su}}}{8}+\frac{n_{\text{mo}}}{4}+\frac{n_{\text{st}}}{2}+\frac{n_{\text{vs}}}{1}},$$

where c is a parameter and $n_{\text{su}},\;n_{\text{mo}},\;n_{\text{st}}$ and $n_{\text{vs}}$ are the numbers of supporting, moderate, strong, and very strong lines of evidence, respectively. Equation (1) specifies that one line of very strong evidence equals two lines of strong, four lines of moderate and eight lines of supporting evidence.

Additionally, the value of ${\text{LR}}^{+}$ for one line of supporting, moderate, strong, and very strong evidence equals $\sqrt[{8}]{c},\;\sqrt[{4}]{c},\;\sqrt{c}$ and c, respectively. This can be interpreted as a point-based system in which points are measured on the $\text{log}\;{\text{LR}}^{+}$ scale and supporting, moderate, strong, and very strong evidence of pathogenicity/benignity are designated ±1, ±2, ±4, ±8 points, respectively, with positive points indicating pathogenic evidence and negative points denoting benign evidence [52]. Under this interpretation, it is straightforward to incorporate intermediate evidence strengths between those established (e.g., ±3 points).

If the prior probability of pathogenicity P(Y=1) is known, the parameter c is determined as the smallest number for which the posterior probability of pathogenicity

(2) $$P(Y=1|E=e)=\frac{{\text{LR}}^{+}(e)\cdot P(Y=1)}{\left({\text{LR}}^{+}(e)-1\right)\cdot P(Y=1)+1}$$

satisfies all qualitative criteria from Richards et al. [45]. That is, P(Y=1∣E=e) must reach at least 0.9 for variants classified as likely pathogenic and 0.99 for variants classified as pathogenic. A prior of P(Y=1)=0.1 implies c=350 [51], whereas P(Y=1)=0.044 implies c=1124 [39].

When the available evidence is real-valued, it is necessary to discretize it for compatibility with ACMG/AMP guidelines [45]. Letting S be a real-valued random variable representing a variant’s assay score, we can estimate the local positive likelihood ratio ${\text{lr}}^{+}(s)$ at score s as the density ratio p(s∣Y=1)p(s∣Y=0) and establish score intervals $\left[\tau_{\text{low}},\tau_{\text{high}}\right]$ such that ${\text{lr}}^{+}(s)$ is greater than the required level for any score in the interval [39]. For example, for supporting evidence, it is required that ${\text{lr}}^{+}(s)\geq \sqrt[{8}]{c},\forall s\in \left[\tau_{\text{su}},\tau_{\text{mo}}\right]$, while for moderate evidence, it is required that ${\text{lr}}^{+}(s)\geq \sqrt[{4}]{c},\forall s\in \left[\tau_{\text{mo}},\tau_{\text{st}}\right]$. Considering functional assay as a predictor, ${\text{lr}}^{+}(s)$ can be seen as the slope of the ROC curve at the predictor’s score s [54].

To calibrate *in silico* tools, Pejaver et al. [39] combined the data from all disease genes and used separate nonparametric methods to estimate P(Y=1) and ${\text{lr}}^{+}(s)$. However, this method relies on a relatively large number of clinical control variants and cannot be reliably applied to most individual genes. Stronger assumptions are therefore necessary, leading us to develop a parametric approach to simultaneously estimate P(Y=1) and ${\text{lr}}^{+}(s)$ in a gene-specific manner.

### 2.2 Data

A curated set of 24 functional datasets were collected from MaveDB [12] and directly from recent publications. A typical dataset from MaveDB contains scores measuring the functional effect of a number of variants, including single and multiple nucleotide substitutions, insertions and deletions. These scores can reflect the effect of the variant on a particular function, such as stability or abundance, or reflect the overall functional fitness of the variant, and datasets frequently contain score measurements for biological or technical replicates. Additionally, some functional datasets include the authors’ categorical interpretation of each variant, annotating them as functionally normal, indeterminate, or functionally abnormal.

In this study, single-nucleotide variants were collected from ClinVar (accessed 09/17/2024) [26]. Subsets of variants, referred to as ClinVar P/LP, ClinVar B/LB, and ClinVar VUS, were constructed by selecting variants with review status of at least one star and clinical significance of pathogenic (P) or likely pathogenic (LP), benign (B) or likely benign (LB), and uncertain significance (VUS), respectively. Single-nucleotide variants in gnomAD genomes and exomes (v4.1.0) [25] were collected, excluding filtered sites using GATK (v4.4.0.0) [31]. Missense variants in each experimental dataset were labeled to indicate their presence in the ClinVar P/LP, B/LB, VUS, and gnomAD sets, and synonymous variants were labeled as such. The intersections of each dataset with the ClinVar and gnomAD sets were constructed by matching variants on the most precise description available, their consequence relative to the GRCh38 genome build, transcript, or protein sequence.

For assays that do not detect splice-altering effects of variants, any variant with a SpliceAI score [21], using the maximum of the acceptor loss/gain and donor loss/gain predictions, greater than a strict threshold of 0.5 [56] was removed, as it is unknown if any associated pathogenic effect is mediated by the missense or the splicing change. The maximum SpliceAI score for the given consequence was used when filtering datasets in which variants are only described at the protein level. For datasets with multiple replicate scores reported, the average score was computed. Author-reported scores were collected without further normalization, excluding the TP53 Transcriptional Assay dataset [23]. For this dataset, the median score across the eight promoters was computed, as done by Fortuno et al. [14], and median scores were normalized to the B/LB variant score distribution. Details on all datasets can be found in Table 1 and in the Supplement.

### 2.3 Skew normal distributions

Assay scores are modeled using skew normal distributions. Under the distribution’s canonical parameterization [2], the probability density function of a random variable X~SN(μ,ω,λ) is given by

$$\text{SN}\left(x;\mu ,\omega ,\lambda \right)=\frac{2}{\omega }\phi \left(\frac{x-\mu }{\omega }\right)\Phi \left(\lambda \left(\frac{x-\mu }{\omega }\right)\right),$$

where μ,ω, and λ denote the location, scale and skew of the distribution, and ϕ and Φ are the probability density and cumulative distribution functions of the standard Gaussian distribution, respectively. Under the alternate parameterization, defined by parameters Δ and Γ, used in the parameter updates of the optimization algorithm, X~SN(μ,ω,λ) can be expressed as $X=\mu +\Delta \cdot T+\Gamma^{\frac{1}{2}}\cdot U$, where T is a random variable from the standard normal distribution truncated below 0 and U is a random variable from the standard normal distribution [27, 40]. Canonical and alternate parameterizations are related through equations in Table 1 of [40], and are included in Supplementary Table S1 for completeness.

Skew normal distributions were chosen as they are able to model the asymmetry observed in functional datasets and have been found to be tractably incorporated into multi-sample mixture models [40].

### 2.4 Modeling

Assay score distributions from each experimental dataset are approximated using a multi-sample two-component skew normal mixture model. Let $S_{P}$ be the set of assay scores for variants in the dataset’s pathogenic and likely pathogenic (P/LP) sample, let $S_{B}$ be the set of assay scores for variants in the dataset’s benign and likely benign (B/LB) sample, and let $S_{G}$ be the set of assay scores for variants in the dataset’s gnomAD sample, used here as a reference sample. The score distribution of each of these random variables is modeled as a two-component skew normal mixture representing functionally abnormal (a) and functionally normal (n) variant assay scores parameterized by $\theta_{a}=\left(\mu^{\left(a\right)},\omega^{\left(a\right)},\lambda^{\left(a\right)}\right)$ and $\theta_{n}=\left(\mu^{\left(n\right)},\omega^{\left(n\right)},\lambda^{\left(n\right)}\right)$, respectively. The mixture model approximating the distributions of $S_{P},\;S_{B}$, and $S_{G}$ can therefore be expressed as

(3) $$p_{S_{i}}\left(s;w_{S_{i}},\theta_{a},\theta_{n}\right)=w_{S_{i}}\cdot \text{SN}\left(s;\theta_{a}\right)+\left(1-w_{S_{i}}\right)\cdot \text{SN}\left(s;\theta_{n}\right),$$

where i∈{P,B,G}.

The local positive likelihood ratio of the model is defined as

$${\text{lr}}^{+}\left(s;w_{S_{P}},w_{S_{B}},\theta_{a},\theta_{n}\right)=\frac{p_{S_{P}}\left(s;w_{S_{P}},\theta_{a},\theta_{n}\right)}{p_{S_{B}}\left(s;w_{S_{B}},\theta_{a},\theta_{n}\right)}.$$

The prior probability of pathogenicity P(Y=1) can be obtained by expressing the reference gnomAD distribution as a two-component mixture of pathogenic and benign score distributions

$$S_{G}\sim p\left(s\right)=P\left(Y=1\right)\cdot p\left(s|Y=1\right)+P\left(Y=0\right)\cdot p\left(s|Y=0\right),$$

and then rearranging Eq. (3) to obtain

(4) $$P\left(Y=1\right)=\frac{w_{S_{G}}-w_{S_{B}}}{w_{S_{P}}-w_{S_{B}}}.$$

Finally, if provided, the sample of synonymous variant assay scores is also modeled as a mixture of the functionally abnormal and functionally normal score distributions to aid in the optimization,

$$p_{S_{S}}\left(s;w_{S_{S}},\theta_{a},\theta_{n}\right)=w_{S_{S}}\cdot \text{SN}\left(s;\theta_{a}\right)+\left(1-w_{S_{S}}\right)\cdot \text{SN}\left(s;\theta_{n}\right).$$

Synonymous variant scores were available for 11 of 24 datasets (Table 1).

#### 2.4.1 Optimization

The parameters of our multi-sample skew normal mixture model can be jointly learned by adapting the expectation-maximization algorithm derived by Peng et al. [40]. With ¨ and ¯ denoting new and old parameter values, respectively, the parameter update equations can be expressed as

$${\ddot{\mu }}^{\left(*\right)}=\frac{\sum_{S_{i}\in \left\{S_{P},S_{B},S_{G},S_{S}\right\}}\sum_{s_{ij}\in S_{i}}{\overset{‾}{p}}_{S_{i}}^{\left(*\right)}\left(s_{ij}\right)\cdot {\overset{‾}{m}}^{\left(*\right)}\left(s_{ij},{\overset{‾}{\Delta }}^{\left(*\right)}\right)}{\sum_{S_{i}\in \left\{S_{P},S_{B},S_{G},S_{S}\right\}}\sum_{s_{ij}\in S_{i}}{\overset{‾}{p}}_{S_{i}}^{\left(*\right)}\left(s_{ij}\right)}\;{\ddot{\Delta }}^{\left(*\right)}=\frac{\sum_{S_{i}\in \left\{S_{P},S_{B},S_{G},S_{S}\right\}}\sum_{s_{ij}\in S_{i}}{\overset{‾}{p}}_{S_{i}}\left(s_{ij}\right){\overset{‾}{d}}^{\left(*\right)}\left(s_{ij},{\ddot{\mu }}^{\left(*\right)}\right)}{\sum_{S_{i}\in \left\{S_{P},S_{B},S_{G},S_{S}\right\}}\sum_{s_{ij}\in S_{i}}{\overset{‾}{p}}_{S_{i}}^{\left(*\right)}\left(s_{ij}\right)}\;{\ddot{\Gamma }}^{\left(*\right)}=\frac{\sum_{S_{i}\in \left\{S_{P},S_{B},S_{G},S_{S}\right\}}\sum_{s_{ij}\in S_{i}}{\overset{‾}{p}}_{S_{i}}^{\left(*\right)}\left(s_{ij}\right){\overset{‾}{g}}^{\left(*\right)}\left(s_{ij},{\ddot{\mu }}^{\left(*\right)},{\ddot{\Delta }}^{\left(*\right)}\right)}{\sum_{S_{i}\in \left\{S_{P},S_{B},S_{G},S_{S}\right\}}\sum_{s_{ij}\in S_{i}}{\overset{‾}{p}}_{S_{i}}^{\left(*\right)}\left(s_{ij}\right)}\;{\ddot{w}}_{S_{i}}=\sum_{s_{ij}\in S_{i}}{\overset{‾}{p}}_{S_{i}}^{\left(*\right)}\left(s_{ij}\right),\forall i\in \{P,B,G,S\}$$

with *=a or $n.\;{\overset{‾}{p}}_{S_{i}}^{\left(a\right)}$ and ${\overset{‾}{p}}_{S_{i}}^{\left(n\right)}$ are defined as ${\overset{‾}{p}}_{S_{i}}^{\left(a\right)}(s)=\frac{{\overset{‾}{w}}_{S_{i}}\cdot \text{SN}\left(s;{\overset{‾}{\theta }}_{a}\right)}{{\overset{‾}{w}}_{S_{i}}\cdot \text{SN}\left(s;{\overset{‾}{\theta }}_{a}\right)+\left(1-{\overset{‾}{w}}_{S_{i}}\right)\cdot \text{SN}\left(s;{\overset{‾}{\theta }}_{n}\right)}$ and ${\overset{‾}{p}}_{S_{i}}^{\left(n\right)}(s)=1-{\overset{‾}{p}}_{S_{i}}^{\left(a\right)}(s)\cdot {\overset{‾}{m}}^{\left(*\right)}(s,\Delta ),\overset{‾}{d}(s,\mu )$, and ${\overset{‾}{g}}^{\left(*\right)}(s,\mu ,\Delta )$ are defined in Supplementary Table S2.

#### 2.4.2 Density constraint

We enforce a monotonicity constraint on the density ratio SNs;θaSNs;θn throughout the optimization. Using the convention of lower scores indicating functionally abnormal variants and higher scores indicating functionally normal variants, this constraint can be stated formally as $\forall s_{1},s_{2}$ such that $s_{1}\leq s_{2}$ implies SNs1;θaSNs1;θn≥SNs2;θaSNs2;θn. Enforcing this constraint alongside the assumption that pathogenic variants are more likely to be functionally abnormal than benign variants, verified during assay validation [6], is sufficient for the positive likelihood ratio of our model to be non-increasing.

**Theorem 1.** *Given a monotonic density ratio* SNs;θaSNs;θnandwSP>wSB,p(s∣Y=1)p(s∣Y=0) *and* P(Y=1∣s) *are also monotonic*.

The proof is in the Supplement. This theorem guarantees that stronger assay scores always lead to stronger evidence of pathogenicity. As is done by Peng et al. [41], a binary search is used to maintain this constraint at each update to the parameters $\mu^{\left(a\right)},\;\Delta^{\left(a\right)},\;\Gamma^{\left(a\right)},\;\mu^{\left(n\right)},\;\Delta^{\left(n\right)}$, and $\Gamma^{\left(n\right)}$.

#### 2.4.3 Initialization

To initialize the parameters of the skew-normal distributions, $\theta_{a}$ and $\theta_{n}$, K-means clustering (K=2) is fit on all observations from our samples. Normal distributions are fit to the sets of observations in each of the two clusters to obtain $\mu^{\left(a\right)},\;\omega^{\left(a\right)},\;\mu^{\left(n\right)}$ and $\omega^{\left(n\right)}$. As it has been observed empirically that the direction of the skew does not change during the iterations of the EM algorithm [40], and to ensure a diverse range of initializations, $\lambda^{\left(a\right)}$ and $\lambda^{\left(n\right)}$ are each sampled randomly from the uniform distribution U(-0.25,0.25). To ensure the initial parameters satisfy the density constraint, checked within the range of our observations, the locations of each component are iteratively moved in opposing directions until $\theta_{a}$ and $\theta_{n}$ satisfy the constraint. To mitigate the effects of random parameter initialization, we performed 100 independent runs of the modeling procedure, retaining the model that achieved the highest likelihood.

#### 2.4.4 Score thresholds and uncertainty quantification

To quantify uncertainty and obtain conservative evidence strength estimates, the modeling procedure is repeated 5,000 times for each dataset on bootstrapped [10] samples of the data, ensuring the P/LP, B/LB, gnomAD, and synonymous observation sets are disjoint. Assay score thresholds for each bootstrap iteration are calculated with respect to the prior probability of pathogenicity estimated by the model in the iteration. Score thresholds are aggregated across bootstrap iterations, using the 5^th^ and 95^th^ percentiles as the final score thresholds defining each pathogenic and benign evidence strength, respectively. An assay reaches a given evidence strength if at least 95% of the bootstrap iterations reach that strength. Models for which bootstrapping leads to invalid prior estimates are excluded when aggregating thresholds.

## 3 Results

**Figure 1:**
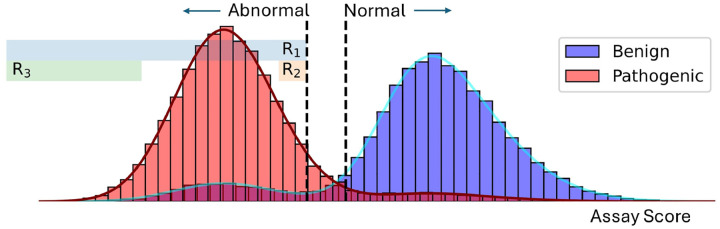
Illustrating the current use of functional data in clinical variant classification. ACMG/AMP recommendations rely on thresholds defining functionally normal and functionally abnormal variants given a set of experimental scores. The evidence strength for variants exceeding these thresholds is computed using pathogenic (red) and benign (blue) control sets. The pathogenic evidence strength is computed as the ratio of true positive rate and false positive rates in region ${\text{R}}_{1}$. As functional data appear as continuous scores, as opposed to binary functional labels, assigning evidence strengths at the variant level can result in more accurate use of functional evidence. Variants with scores in region ${\text{R}}_{3}$, far below the threshold, should be assigned higher evidence strengths than those assigned to variants with scores in region ${\text{R}}_{2}$, slightly below the threshold.

Functional data are one of the few lines able to serve as very strong evidence of pathogenicity. Brnich et al. [6] refined the use of functional data in variant classification, recommending steps to assess the clinical validity of functional data and proposing a rule-based system for assigning evidence strengths. In this system, the assay score thresholds that define functionally normal and abnormal variants are determined using a set of controls. These thresholds are used to categorize variants, conventionally as functionally normal, functionally abnormal, or indeterminate, and the evidence strength assignment for all variants exceeding these thresholds is computed using global positive likelihood ratio; i.e., the ratio of true positive rate to false positive rate in a score interval. Assigning evidence strengths in this manner for well-behaved assays overestimates the strength of functional evidence for variants scoring near the thresholds and underestimates the strength of evidence for variants far exceeding the thresholds, as illustrated in Figure 1. This motivates the need for evidence strengths to be assigned at a variant level, taking into consideration the variant’s raw (continuous) assay score. Assigning evidence strengths at the variant level, using the local positive likelihood ratio, should result in more reliable evidence assignments and more accurate variant classification.

### 3.1 The model captures a variety of assay score distributions

**Figure 2:**
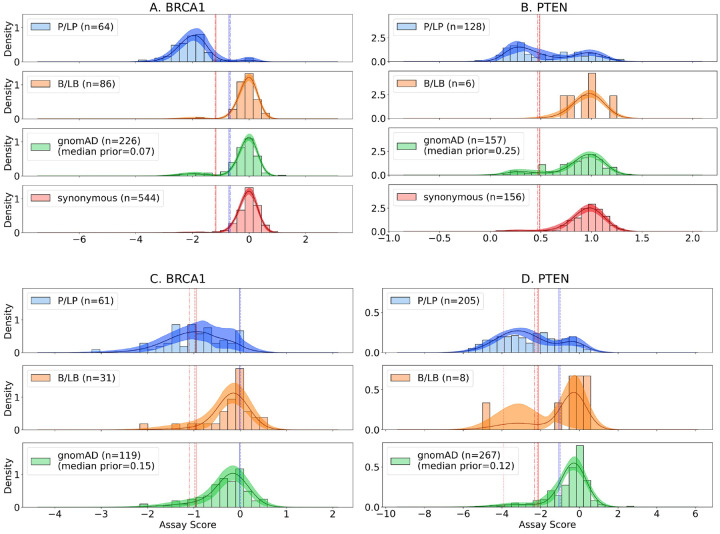
Example model fits to four functional datasets, assessing the effect of BRCA1 variants (panels A and C; left) and PTEN variants (panels B and D; right). Datasets for panels A, B, C, and D were taken from Findlay at al. [13], Matreyek et al. [30], Adamovich et al. [1], and Mighell et al. [32], respectively. Median density across all iterations is displayed along with the estimated 95% confidence interval. Score thresholds corresponding to each pathogenic and benign evidence strength reached by the model are plotted in red and blue, respectively, with supporting, moderate, strong, and very strong displayed as solid, dashed, dashed-dotted, and dotted lines, respectively. Differences in the gnomAD variants included in each dataset contribute to the differences in prior probability estimates.

To demonstrate the utility of our calibration method, we visualized the distributions and score thresholds given by the model on four datasets in Figure 2. The learned models capture the distributions of the data and justify the selection of the skew normal family. Figure 2A shows that the calibration model assigns pathogenic and benign evidence to functional datasets in which the assay separates the P/LP and B/LB distributions. In contrast, Figure 2B demonstrates the calibration model appropriately does not assign benign evidence to variants with mechanisms of pathogenicity not captured by the particular assay. This reflects limitations of the VAMP-seq assay which assesses the impact on protein stability and does not measure all types of functional impacts such as enzymatic activity [33], thus leaving a significant proportion of pathogenic variants with scores that are similar to those of synonymous variants [30]. This calibration method based on skew normal models reflects what we intuitively understand about the strengths and limitations of these assays.

**Figure 3:**
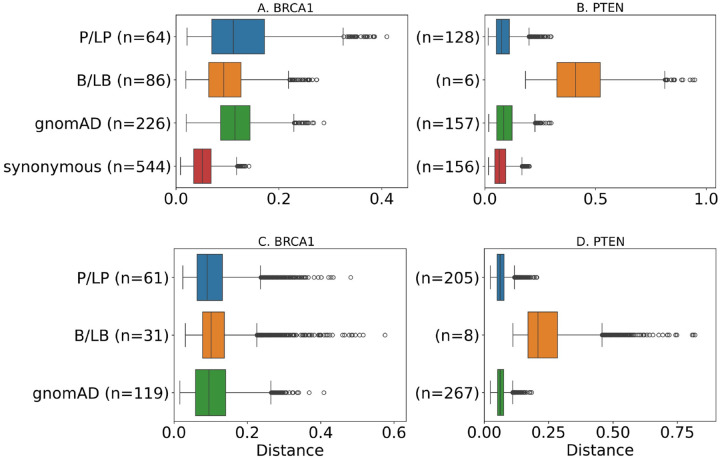
Distributions of normalized distances between empirical and estimated cumulative distribution functions for each sample, obtained using 5,000 bootstrapping iterations, for the same datasets as in Figure 2. Datasets for panels A, B, C, and D were taken from Findlay at al. [13], Matreyek et al. [30], Adamovich et al. [1], and Mighell et al. [32], respectively.

To quantify the quality of the model fit, for each bootstrap iteration, the normalized Yang et al. [59] distance (p=2) of the empirical and learned cumulative distribution functions were computed for the P/LP, B/LB, gnomAD, and synonymous samples. The distance function gives values between 0 and 1 and is computed using real-valued vectors at all unique scores in the assay. Figure 3 visualizes the distribution of distances across the 5,000 bootstrap iterations for each sample. The distances computed on the gnomAD and synonymous samples can serve as a metric to decide whether to accept the model fit. Empirically, we observed the median distance computed on these two samples was typically below 0.2. Being smaller and potentially accumulated in a biased fashion, the distances computed on the P/LP and B/LB samples are generally larger and not as reliable in evaluating the quality of the model fit. Analysis of additional datasets from Table 1 is presented in the Supplement.

### 3.2 Variant-level evidence strengths vs. author-provided annotations

**Figure 4:**
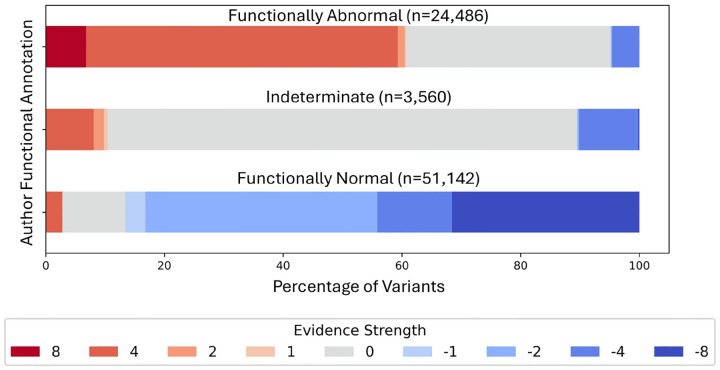
Distribution of evidence strengths assigned by our method for all variants with functional annotations provided by the authors of experimental studies (Table 1). Author annotations were harmonized to a consistent vocabulary. Annotations of “enriched” from a single dataset, Lo et al. [28], were excluded due to the limited number of variants.

We compared the distributions of variant-specific evidence strengths assigned by the calibration model to the functional annotations provided by the authors (Figure 4). Evidence strengths were assigned in an out-of-bag manner; i.e., the mode of the strengths assigned to each variant by all models for which the variant was not used to fit. Of the variants annotated by the study authors as functionally abnormal, 60% were assigned pathogenic evidence of some strength, and 87% of variants annotated as functionally normal were assigned benign evidence of some strength by our model. These reductions are consistent with the expectation that discretizing assay scores prior to calculating evidence strengths results in an overestimation of evidence strengths. Fifty-two percent of variants annotated as functionally abnormal by authors reached the level of pathogenic strong (+4), and 7% of variants annotated as functionally abnormal reached the level of pathogenic very strong (+8) using the model, exceeding the default strength specified in Richards et al. [45]. Thirty-two percent of variants annotated by authors as functionally normal reached benign very strong (−8), exceeding the current default strength of benign strong (−4).

### 3.3 Variant-level evidence improves clinical variant classification

**Figure 5:**
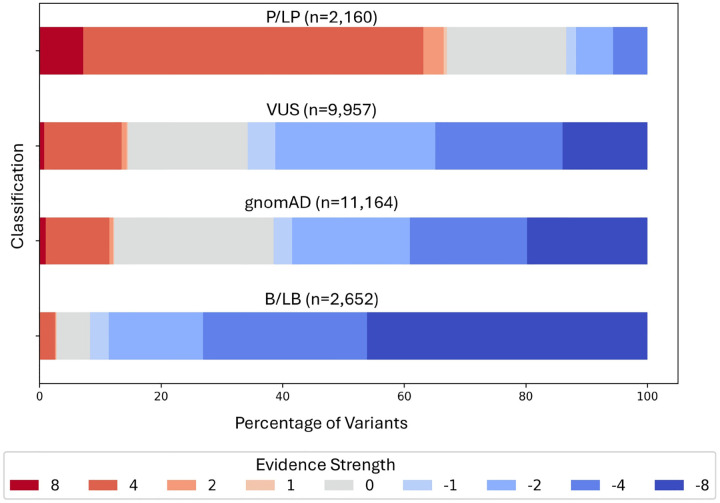
Distribution of evidence strengths assigned by our method for variants in the P/LP, VUS, gnomAD, and B/LB samples (each dataset was considered independent of each other). The results suggest the need for reclassification of ClinVar variants using updated functional evidence.

The out-of-bag evidence strength distributions for variants present in ClinVar and gnomAD are visualized in Figure 5. Of the 2, 160 P/LP variants, 67% were assigned pathogenic evidence strengths, and 92% of the 2, 652 B/LB variants were assigned benign evidence strengths. Eighty-one percent of the 9,957 VUS were assigned either pathogenic or benign evidence strengths, with 66% assigned benign evidence strengths and 15% assigned pathogenic evidence strengths, broadly consistent with previous experience [18].

**Figure 6:**
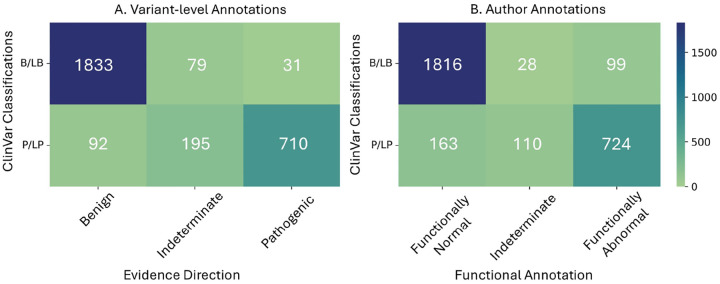
Comparison of our variant-level functional annotations, Figure 6A, to author-provided annotations, Figure 6B, for the subset of ClinVar variants in author-annotated scoresets. Variant-level evidence assignments less than 0 were labeled with benign evidence direction, assignments greater than 0 were labeled with pathogenic evidence direction, and 0 was considered indeterminate.

To demonstrate the impact this calibration method could have on variant classification, we categorized our variant-level evidence strength assignments based on the direction of evidence and compared these to available author-provided functional annotations on the subset of variants with P/LP or B/LB ClinVar classifications. Figure 6 shows the number of P/LP and B/LB variants in ClinVar that were assigned pathogenic or benign evidence directionality (Figure 6A) vs. the number of variants that were assigned author-provided functional annotations (Figure 6B). We summarized these results by calculating the global positive likelihood ratio (${\text{LR}}^{+}$) and diagnostic odds ratio (DOR) for our method vs. author-provided functional annotations. To do so, we first dichotomized the three-way annotations for our method as pathogenic vs. non-pathogenic and obtained ${\text{LR}}^{+}=\frac{710}{710+195+92}\cdot \frac{31+79+1833}{31}=44.6$. We then dichotomized the three-way author-provided annotations as functionally abnormal vs. the rest and obtained ${\text{LR}}^{+}=\frac{724}{724+110+163}\cdot \frac{99+28+1816}{99}=14.3$. Furthermore, our variant-level annotations resulted in DOR = 152.6 compared to DOR = 49.4 for the author-provided annotations. Similarly, by dichotomizing our annotations into benign vs. non-benign, we obtained ${\text{LR}}^{+}=10.2$ and DOR = 163.9 for predicting benignity, compared to the author-provided annotations where we obtained ${\text{LR}}^{+}=5.7$ and DOR = 72.6 by dichotomizing the annotations as functionally normal vs. the rest. These results provide evidence that our variant-level calibration method is substantially more accurate than author-provided annotations and provide a basis for improved variant classification.

## 4 Related Work

To our knowledge, this work presents the first calibration approach for high-throughput functional assays for clinical utility. However, model calibration is a well-known issue in machine learning, where the objective is to adjust the scores of trained classifiers to properly reflect posterior probabilities [42, 34, 24]. Typical algorithms in this space rely on relatively large quantities of data and, more importantly, do not assume a bias model. This is critical, as the balance of pathogenic and benign variants in ClinVar is heavily skewed towards pathogenic. Therefore, we introduced a reference set (gnomAD) whose primary purpose was to help estimate the class prior, thus performing calibration in a semi-supervised manner.

Calibration has also been studied in the context of variant interpretation [3]. Pejaver et al. [39] proposed a nonparametric method for the calibration of computational tools, but this model relies on larger quantities of control variants (e.g., known P/LP or B/LB), which are unavailable for most genes. Therefore, stronger assumptions must be adopted to constrain the calibration. In terms of high-throughput functional data, assay score distributions have previously been modeled with multi-sample Gaussian mixtures (MSGMM), learned in the positive-unlabeled classification setting to assign discrete functional annotations to variants [38]. Although not addressed in that work, the distributions learned by the MSGMM can be used to calculate a variant’s posterior probability of being functionally abnormal. However, in the absence of overly restrictive assumptions on the functional mechanisms of disease, these probabilities do not contain sufficient information to calibrate assay scores to posterior probabilities of pathogenicity. The MSGMM method also does not impose density constraints on the component distributions and, therefore, cannot guarantee the monotonicity of the posterior. An additional method, MaveLLR, has been proposed to quantify the evidence strength of assay scores [55]. This method approximates the assay score distributions of pathogenic and benign variants using kernel density estimation (KDE). These density functions are used to compute the local positive likelihood ratio for each assay score. Along with the sensitivity of KDE to the chosen kernel in the presence of small data, this approach is not capable of estimating the prior probability of pathogenicity, a value critical to calibration. These limitations prevent rigorous calibration and reliable evidence strength assignments.

## 5 Discussion

High-throughput functional assays are a valuable tool in the clinical classification of variants [20]. To ensure consistent and accurate application of functional evidence, we present a statistical framework for calibrating high-throughput functional assays. This framework jointly models assay score distributions of pathogenic and benign variants, along with that of a reference set of variants from gnomAD, as unique mixtures of two skew normal distributions. This calibration model computes the local positive likelihood ratio along with the prior probability of pathogenicity for variants in a gene or domain.

The results demonstrate suitability of skew normal mixture models to faithfully capture assay score distributions from a diverse set of high-throughput functional experiments. The calibration model rigorously quantifies the strength of functional evidence in clinical variant classification and its distinct feature is that the evidence strength assigned to variants is variant-specific. Our model thus extends the current standard in the field by allowing multiple, distinct evidence strengths for the same assay [6]. The evidence assigned using this calibration model can be incorporated into the ACMG/AMP evidence-based framework. While the Richards et al. [45] guidelines limit the strength of functional studies to strong and Brnich et al. [6] allow pathogenic evidence to be applied up to very strong, we evaluate the full potential of functional evidence, allowing for assays to additionally serve as very strong benign evidence if the necessary likelihood ratio is met. Then it can be considered if there is additional rationale for decreasing the strength of evidence applied in classification.

We have identified cases in which the calibration model assigns evidence strengths inconsistent with those applied by ClinGen Variant Curation Expert Panels (VCEPs). The PTEN VCEP [29] allows for the lipid phosphatase assay to serve as either moderate pathogenic evidence or supporting benign evidence, whereas our calibration model indicates the assay reaches sufficient positive likelihood ratios necessary to serve as up to very strong pathogenic evidence and moderate benign evidence. The skew normal mixture has a large degree of uncertainty on the benign variant distribution, a result of the bootstrapped samples of eight observations allowing for the overrepresentation of a single benign variant with low activity. The BRCA1 HDR assay is applied by the ENIGMA BRCA1 and BRCA2 Expert Panel [37] as a line of strong benign evidence, while the calibration model assigns a maximum strength of supporting benign evidence for this assay. We recommend further investigation into this case. The ClinGen Criteria Specification Registry for MSH2 (release 8/9/2024) specifies a calibrated functional assay can serve as supporting, moderate, or strong pathogenic evidence and can serve as supporting or strong benign evidence based on the calibrated assay’s ${\text{LR}}^{+}$, with positive likelihood ratio thresholds corresponding to a prior probability of pathogenicity of 0.1. The calibration model approximates the MSH2 Resistance assay [22] to have median $\text{log}\;{\text{LR}}^{+}$ values of −2.05 (95% CI [−2.93, −1.52]) and 5.06 (95% CI [3.37, 10.28]) at the lowest and highest assay scores observed. With an estimated median prior of 2%, the calibration model limits this assay to moderate benign evidence and strong pathogenic evidence.

This calibration approach is limited by its dependence on the availability of an unbiased sample of known pathogenic and benign variants to accurately compute local positive likelihood ratios. The modeling does not account for potential biases in the collection of classified variants; e.g., the possibility of the pathogenic variants being more deleterious and having larger functional effect. Further work is needed to detect and quantify the severity of bias in assay distributions and correct for the effect of this bias on calibration [9, 61, 60]. While we limit circularity in calibrating the BRCA1 dataset [13], functional data (either from the other datasets used here or other independent functional data) may already have been incorporated in classifying the existing set of pathogenic and benign variants. Such detailed application of evidence is inconsistently recorded in ClinVar, making it difficult to fully account for circularity. Thus, future calibration approaches must be aware of potential risks of circularity. Additionally, while the skew normal distributions capture the distributions of the high-throughput functional data analyzed here, the quality of the model fit may vary on other datasets and is affected by the normalization steps taken in generating functional scores. The calibration approach has not been evaluated on genes where molecular mechanisms of disease include both gain and loss of function. A three-component mixture model could be an attractive alternative for cases with multiple molecular mechanisms of disease and such a model could be investigated as functional studies with this pattern accumulate.

Overall, our work demonstrates the feasibility of calibrating high-throughput functional assays for clinical variant classification. We find that the existing assays are sufficiently reliable to contribute to variant classification and thus reduce the number of rapidly accumulating VUS. Finally, calibration of functional assays where scores are treated on a continuous scale, will lead to an increased accuracy of genetic diagnosis and improved medical management for individuals affected by Mendelian disorders.

## Supplementary Material

Supplement 1

## Figures and Tables

**Table 1:** Number of single nucleotide variants with P/LP, B/LB, gnomAD, Synonymous, and VUS labels in data used in this work. A mean score was used for all variants with multiple replicates. To limit circularity in the analysis, as the dataset has contributed to a substantial number of classifications, ClinVar classifications for the BRCA1 Saturation Genome Editing dataset [13] are limited to those submitted prior to the publication (release 05/05/2018). Additionally, as this dataset is mapped to GRCh37 coordinates, gnomAD v2.1.1 was used in place of gnomAD v4.1.0

Gene	Experiment	P/LP	B/LB	gnomAD	Synonymous	VUS
BRCA1	Cisplatin resistance assay [1]	61	31	119	0	150
BRCA1	HDR assay [1]	59	47	158	0	230
BRCA1	Saturation genome editing [13]	64	86	226	544	287
BRCA2	HDR assay [19]	49	45	240	0	101
MSH2	Resistiance assay [22]	114	273	1460	0	1598
TP53	Transcriptional Assay [23]	201	132	555	0	652
TP53	Growth assay- p53 WT [16]	213	136	570	376	667
TP53	Growth assay- p53 NULL [16]	213	136	570	376	667
TP53	Growth assay- etoposide [16]	213	136	570	376	667
PTEN	Lipid phosphatase activity assay [32]	205	8	267	0	685
PTEN	VAMP-seq [30]	128	6	157	156	415
VHL	Saturation genome editing [7]	65	15	357	386	180
BAP1	Saturation genome editing [57]	11	976	1714	0	1145
BRCA1	HDR assay [47]	36	36	269	0	294
BRCA2	Saturation prime editing [11]	5	2	61	106	32
NPC1	Saturation prime editing- HEK293T [11]	28	1	124	228	44
TPK1	Yeast complementation [58]	6	1	302	572	91
TP53	HDR assay [5]	210	136	567	0	665
HMBS	Yeast complementation [55]	52	5	488	306	171
PRKN	VAMP-seq [8]	15	14	849	1	108
OTC	Yeast growth assay [28]	93	10	153	0	82
RAD51C	Saturation genome editing [36]	4	407	900	0	786
MSH2	Mutation rate assessment [35]	34	7	89	0	57
CALM1	Yeast complementation [58]	81	8	401	296	183
