# Supplementary material for: Gene-based calibration of high-throughput functional assays for clinical variant classification
Source: bioRxiv. 2025 May 4:2025.04.29.651326. Preprint. [Version 1] doi: 10.1101/2025.04.29.651326 (PMC12248162; doi:10.1101/2025.04.29.651326)
Supplement: Supplement 1 [file media-1.pdf]

Supplementary Materials

Proof of Monotonicity of the Local Positive Likelihood Ratio

To prove Theorem 1, the local positive likelihood ratio is expressed as

$$\begin{aligned}\mathrm{lr}^+(s; w_{S_P}, w_{S_B}, \theta_a, \theta_n) &= \frac{p_{S_P}(s; w_{S_P}, \theta_a, \theta_n)}{p_{S_B}(s; w_{S_B}, \theta_a, \theta_n)} \\ &= \frac{w_{S_P}\mathrm{SN}(s; \theta_a) + (1 - w_{S_P})\mathrm{SN}(s; \theta_n)}{w_{S_B}\mathrm{SN}(s; \theta_a) + (1 - w_{S_B})\mathrm{SN}(s; \theta_n)}\end{aligned}$$

The derivitive of $\mathrm{lr}^+(s; w_{S_P}, w_{S_B}, \theta_a, \theta_n)$ is computed as

$$\begin{aligned}\frac{\mathrm{d}}{\mathrm{d}s}\mathrm{lr}^+(s; w_{S_P}, w_{S_B}, \theta_a, \theta_n) =& \frac{p_{S_B}(s; w_{S_B}, \theta_a, \theta_n)\left(w_{S_P}\mathrm{SN}^{'}(s; \theta_a) + (1 - w_{S_P})\mathrm{SN}^{'}(s; \theta_n)\right)}{p^2_{S_B}(s; w_{S_B}, \theta_a, \theta_n)} - \\ & \frac{p_{S_P}(s; w_{S_P}, \theta_a, \theta_n)\left(w_{S_B}\mathrm{SN}^{'}(s; \theta_a) + (1 - w_{S_B})\mathrm{SN}^{'}(s; \theta_n)\right)}{p^2_{S_B}(s; w_{S_B}, \theta_a, \theta_n)}\end{aligned}$$

Combining and simplifying terms, this can be expressed as

$$\frac{\mathrm{d}}{\mathrm{d}s}\mathrm{lr}^+(s; w_{S_P}, w_{S_B}, \theta_a, \theta_n) = \frac{(w_{S_P} - w_{S_B})\left(\mathrm{SN}(s; \theta_n)\mathrm{SN}^{'}(s; \theta_a) - \mathrm{SN}(s; \theta_a)\mathrm{SN}^{'}(s; \theta_n)\right)}{p^2_{S_B;}(s; w_{S_B}, \theta_a, \theta_n)}$$

As it is given that $\frac{\mathrm{SN}(s;\theta_a)}{\mathrm{SN}(s;\theta_n)}$ is monotonic, the derivitive can be expressed as

$$\frac{\mathrm{d}}{\mathrm{d}s}\frac{\mathrm{SN}(s; \theta_a)}{\mathrm{SN}(s; \theta_n)} = \frac{\mathrm{SN}(s; \theta_n)\mathrm{SN}^{'}(s; \theta_a) - \mathrm{SN}(s; \theta_a)\mathrm{SN}^{'}(s; \theta_n)}{\mathrm{SN}^2(s; \theta_n)}$$

and this value is positive for all scores s if $\frac{\mathrm{SN}(s;\theta_a)}{\mathrm{SN}(s;\theta_n)}$ is monotonically increasing and negative if monotonically decreasing.

The derivitive of the local positive likelihood ratio can be expressed as

$$\frac{\mathrm{d}}{\mathrm{d}s}\mathrm{lr}^+(s; w_{S_P}, w_{S_B}, \theta_a, \theta_n) = c(s) \cdot \frac{\mathrm{d}}{\mathrm{d}s}\frac{\mathrm{SN}(s; \theta_a)}{\mathrm{SN}(s; \theta_n)}$$

where $c(s) = \frac{w_{S_P} - w_{S_B}}{p^2_{S_B}(s; w_{S_B}, \theta_a, \theta_n)} > 0 \; \forall s$, given $w_{S_P} > w_{S_B}$, from which it follows that the local positive likelihood ratio is monotonic.

To show the posterior is monotonic, the posterior can be expressed using Bayes' Rule as

$$\begin{aligned}P(Y = 1|s) &= \frac{p(s|Y = 1)P(Y = 1)}{p(s|Y = 1)P(Y = 1) + p(s|Y = 0)(1 - P(Y = 1))} \\ &= \frac{1}{1 + \frac{1 - P(Y=1)}{P(Y=1)}\frac{p(s|Y=0)}{p(s|Y=1)}} \\ &= \frac{1}{1 + \frac{1 - P(Y=1)}{P(Y=1)}\mathrm{lr}^+(s; w_{S_P}, w_{S_B}, \theta_a, \theta_n)^{-1}}\end{aligned}$$

The derivitive can be expressed as

$$\frac{\mathrm{d}}{\mathrm{d}s}P(Y=1|s) = \frac{\frac{1-P(Y=1)}{P(Y=1)}\mathrm{lr}^+(s;w_{S_P},w_{S_B},\theta_a,\theta_n)^{-2}\frac{\mathrm{d}}{\mathrm{d}s}lr^+(s;w_{S_P},w_{S_B},\theta_a,\theta_n)}{\left(1+\frac{1-P(Y=1)}{P(Y=1)}\mathrm{lr}^+(s;w_{S_P},w_{S_B},\theta_a,\theta_n)^{-1}\right)^2}$$
$$= c_2(s)\frac{\mathrm{d}}{\mathrm{d}s}lr^+(s;w_{S_P},w_{S_B},\theta_a,\theta_n)$$

with $c_2(s) = \frac{\frac{1-P(Y=1)}{P(Y=1)}}{\mathrm{lr}^+(s;w_{S_P},w_{S_B},\theta_a,\theta_n)^2\left(1+\frac{1-P(Y=1)}{P(Y=1)}\mathrm{lr}^+(s;w_{S_P},w_{S_B},\theta_a,\theta_n)^{-1}\right)^2} > 0\ \forall s$, showing the posterior is monotonic.

Skew Normal Mixture Model Related Quantities and Update Equations

Alternate parameterization		Related quantities
Canonical →Alternate	Alternate → Canonical	
$\Delta = \omega\delta$	$\lambda = \mathrm{sign}(\Delta)\sqrt{\Delta^2/\Gamma}$	$\delta = \lambda/\sqrt{1+\lambda^2}$
$\Gamma = \omega^2 - \Delta^2$	$\omega = \sqrt{\Gamma + \Delta^2}$	

Table S1: Parameterizations of the skew normal distribution

$\bar{m}^{(*)}(s,\Delta) = x - \nu(s,\bar{\theta}^{(*)}) \cdot \Delta$
$\bar{d}^{(*)}(s,\mu) = \nu(s,\bar{\theta}^{(*)}) \cdot (s-\mu)$
$\bar{g}^{(*)}(s,\mu,\Delta) = (x-\mu)^2 - 2\cdot\Delta\cdot\nu(s,\bar{\theta}^{(*)}) + \Delta^2 \cdot w(s,\bar{\theta}^{(*)})$
$\nu(s,\theta) = \mathbb{E}[T_s]$
$w(s,\theta) = \mathbb{E}[T_s^2]$
$T_s \sim \mathrm{TN}(\delta/\omega(s-\mu), 1-\delta^2, \mathbb{R}^+)$

Table S2: Related quantities for EM updates

Complete Results

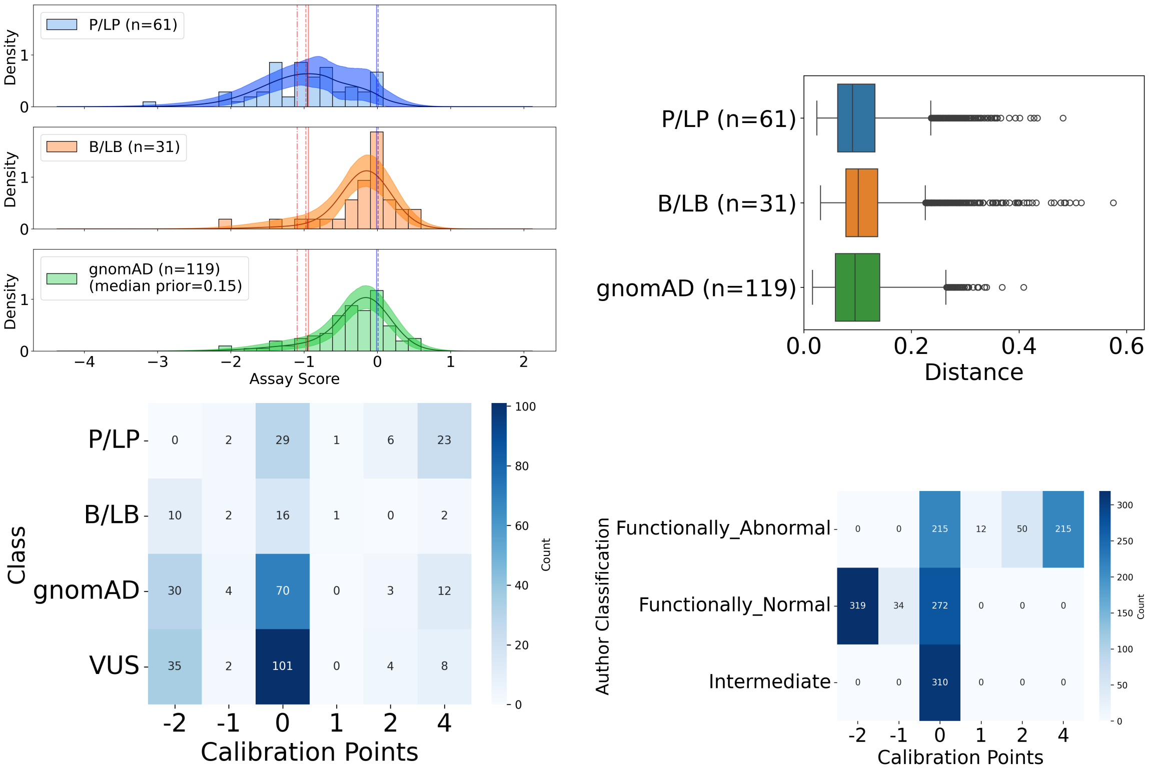

Figure S1: BRCA1- Cisplatin Resistance Assay

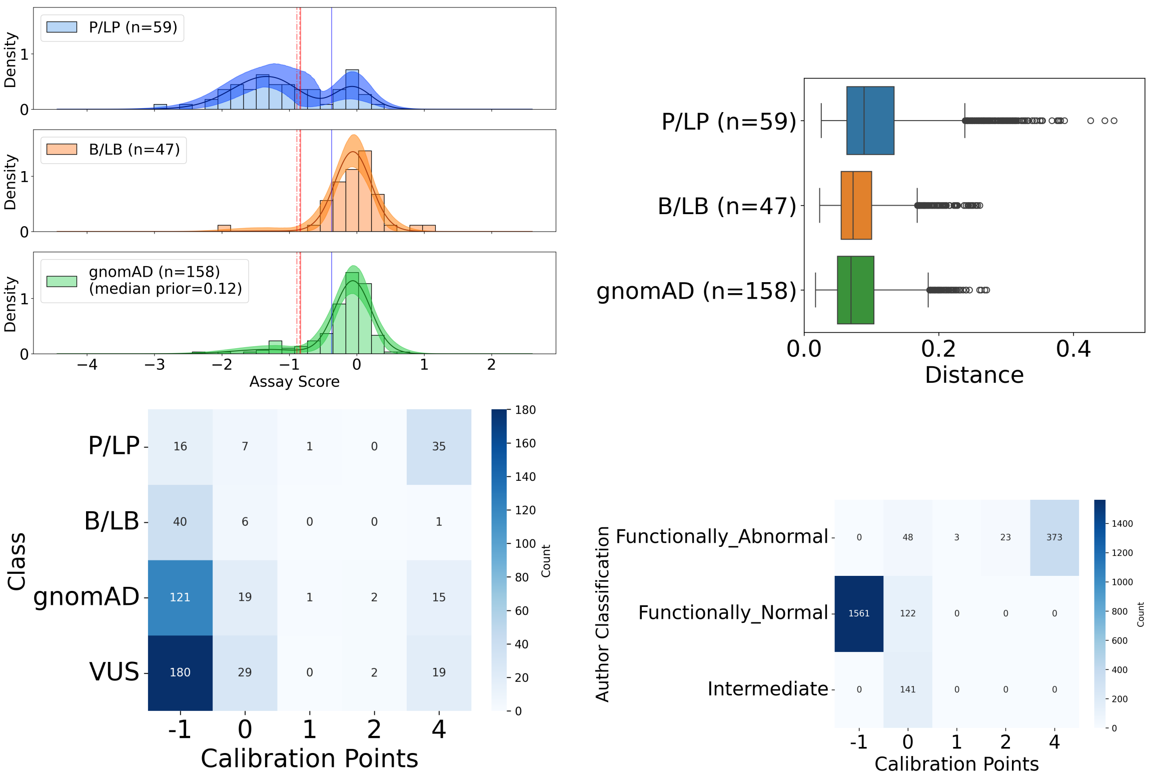

Figure S2: BRCA1- HDR Assay

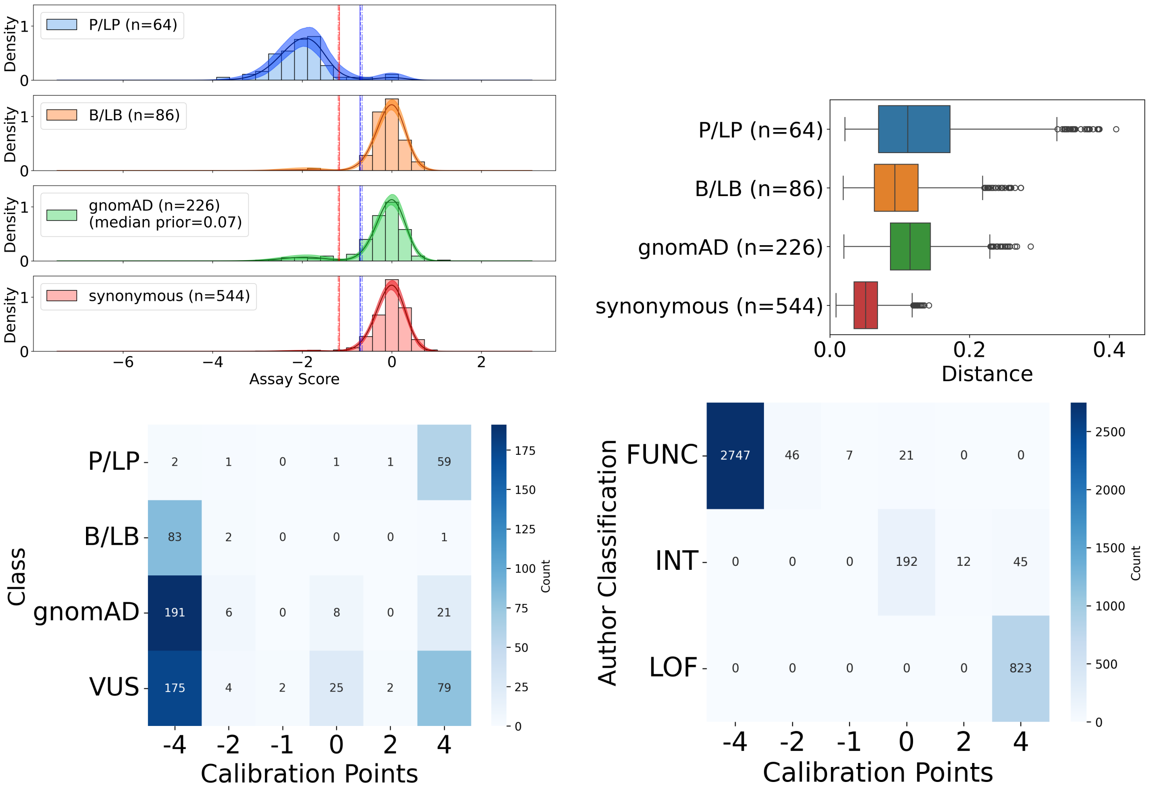

Figure S3: BRCA1- Saturation Genome Editing

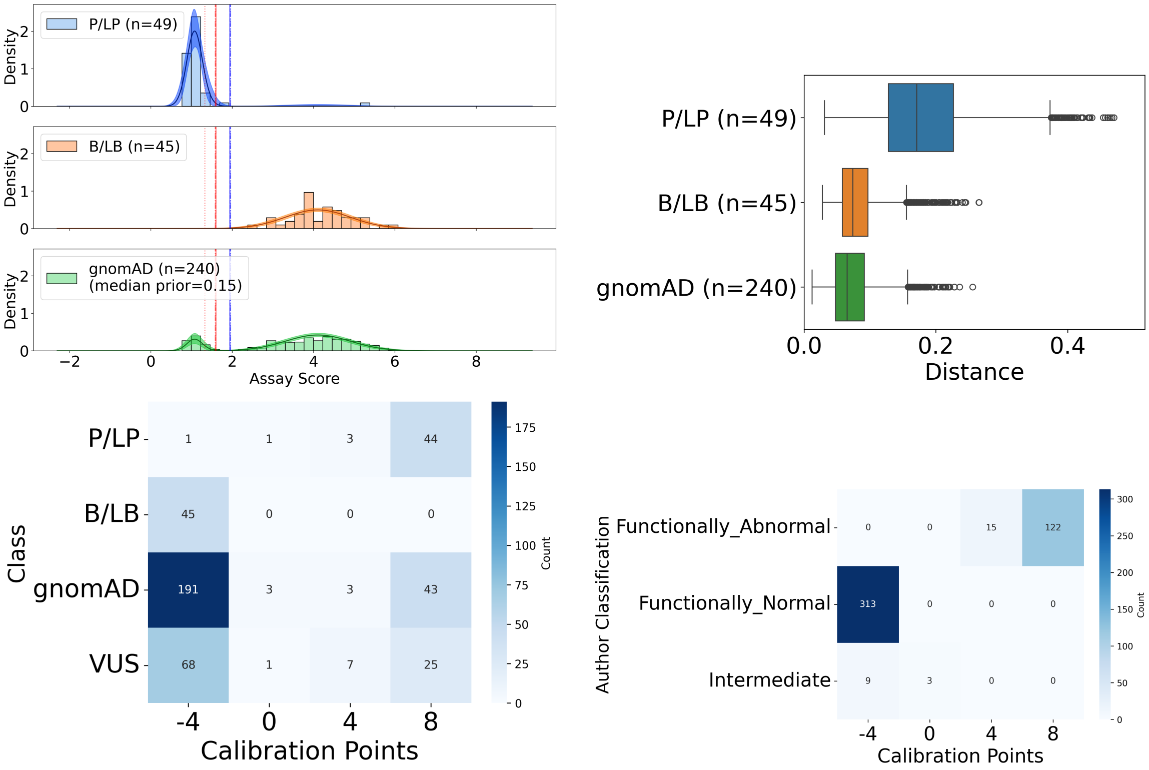

Figure S4: BRCA2- HDR

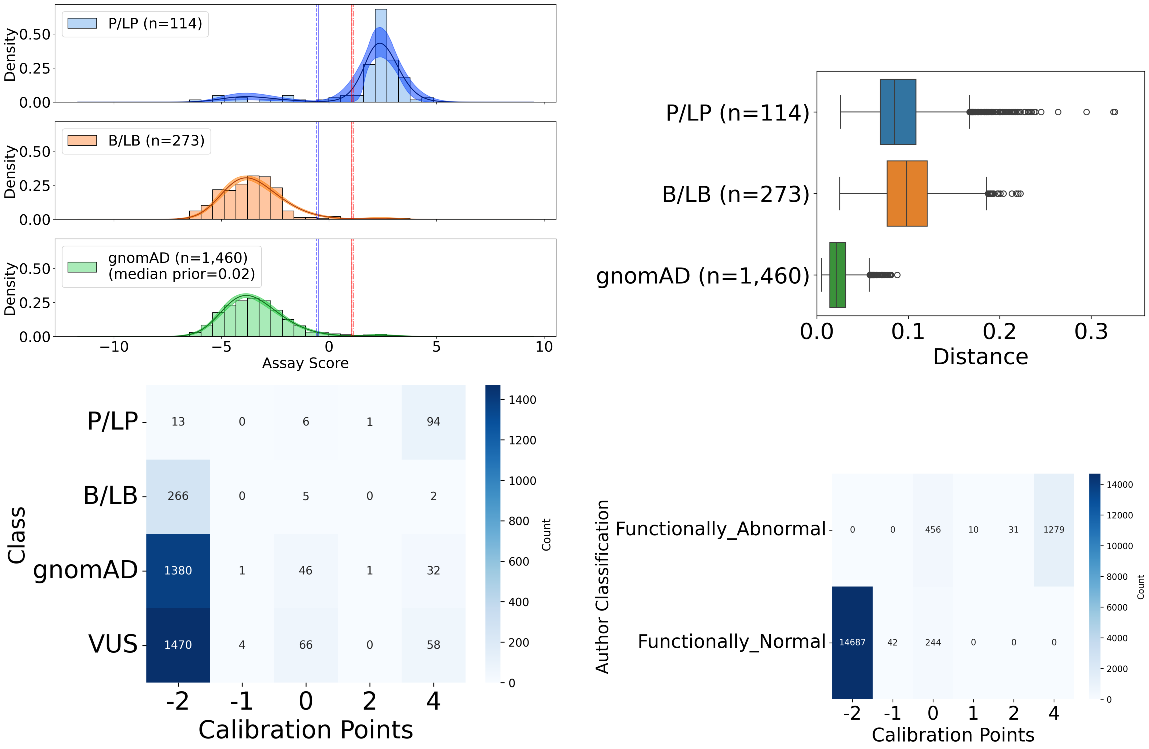

Figure S5: MSH2- Resistance assay

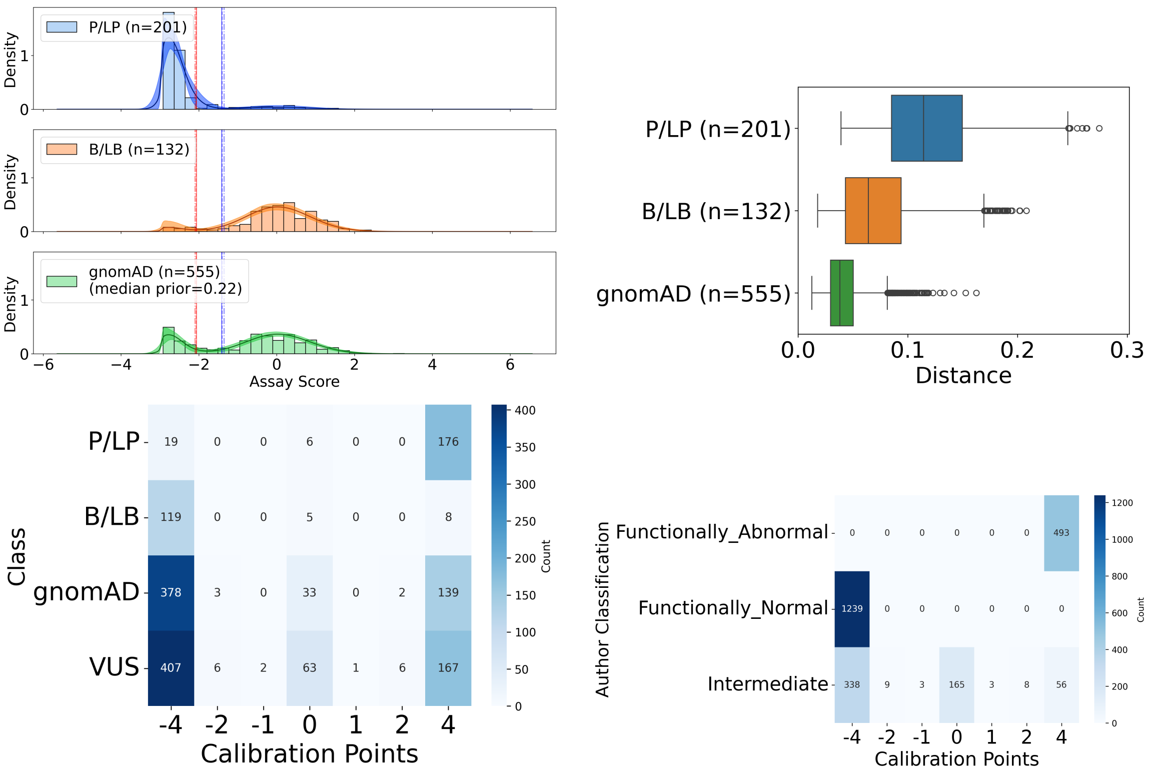

Figure S6: TP53- Transcriptional assay

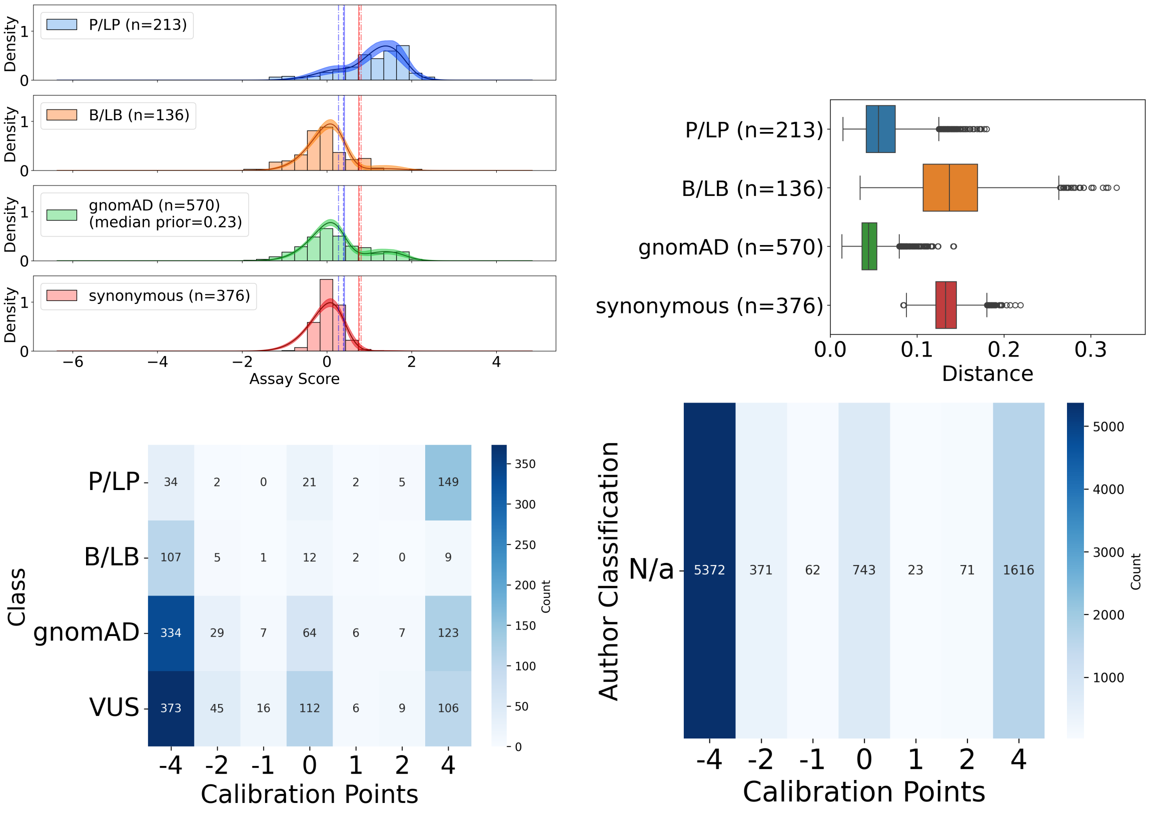

Figure S7: TP53- Growth Assay (p53 WT)

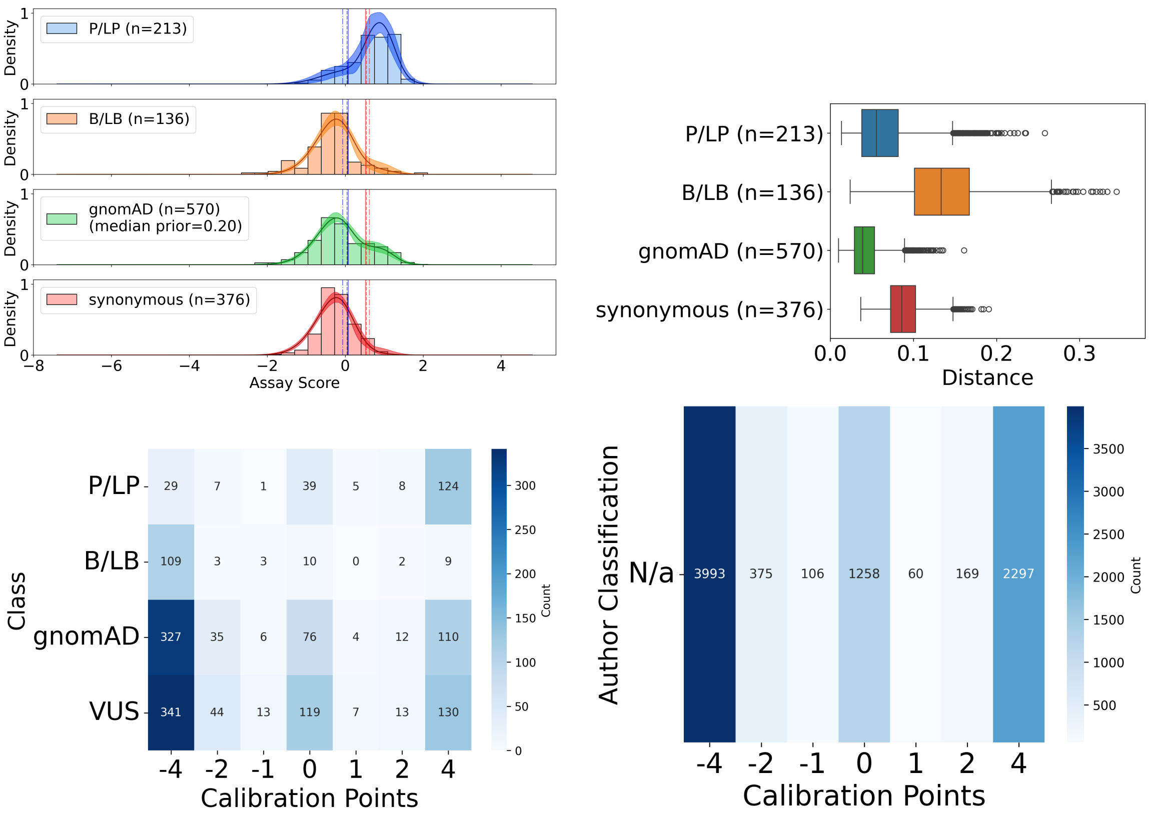

Figure S8: TP53- Growth assay (p53 NULL)

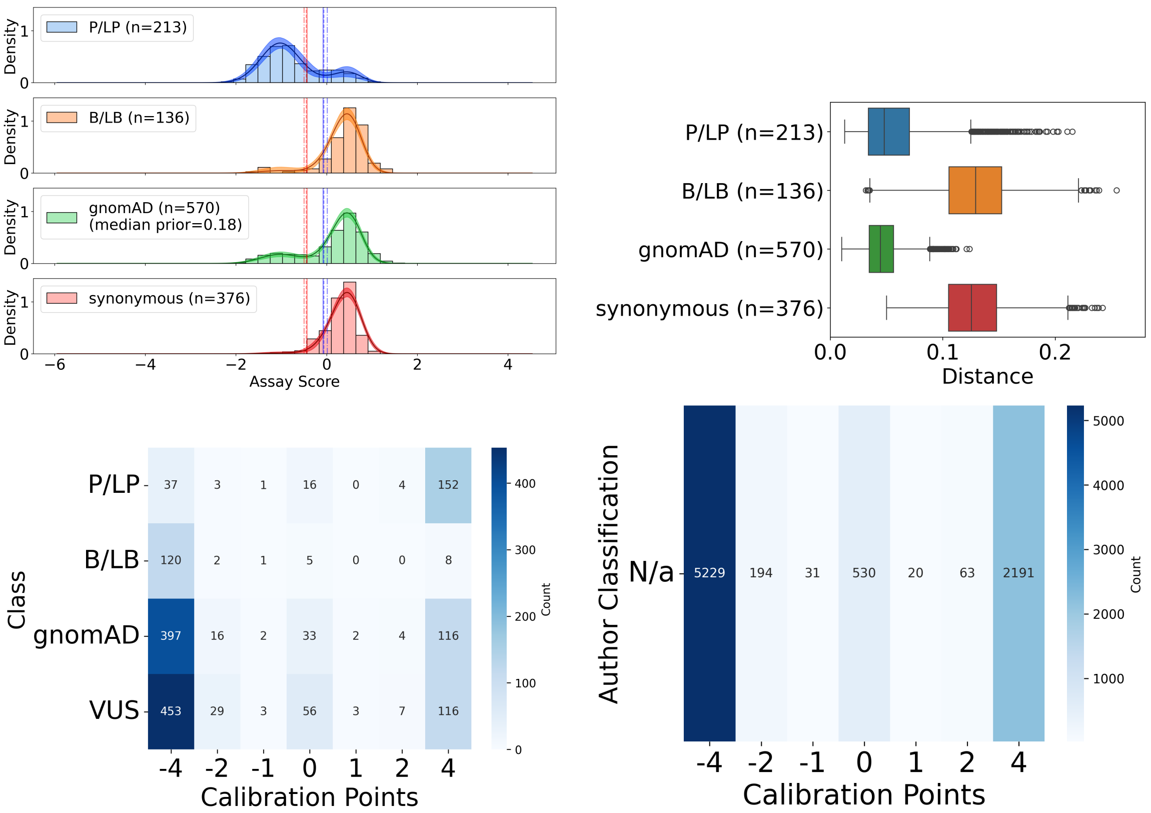

Figure S9: TP53- Growth assay (etoposide)

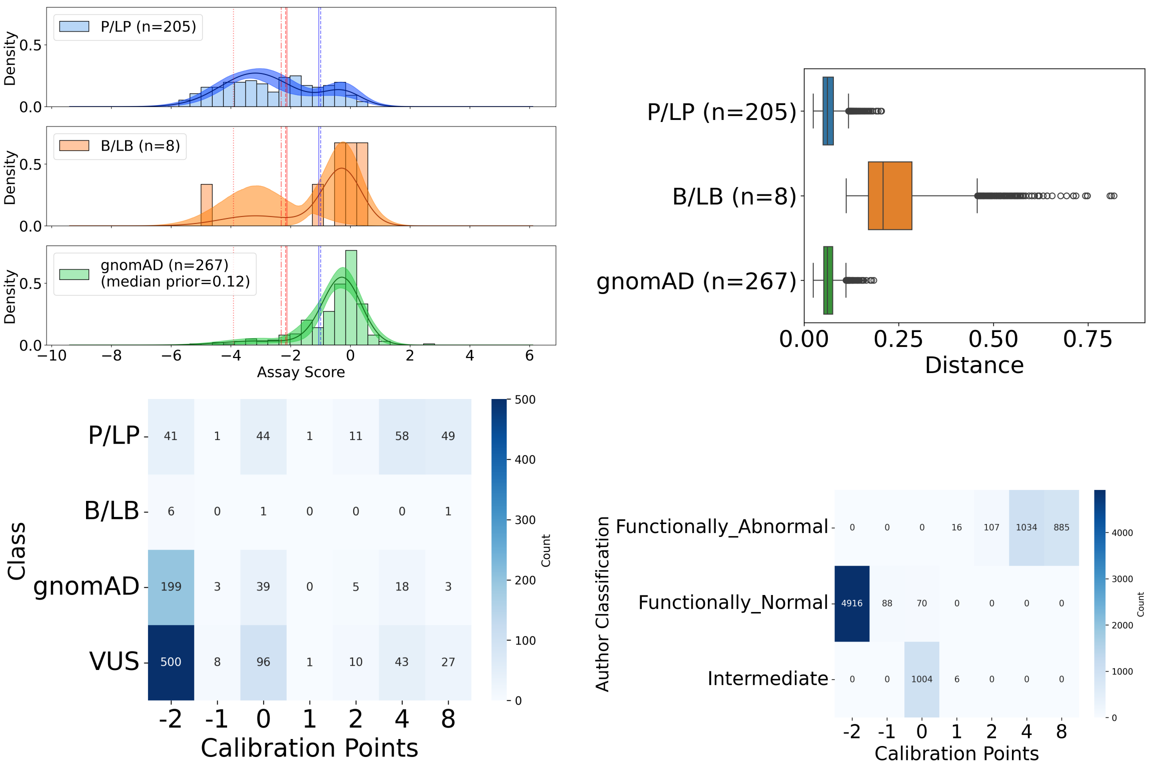

Figure S10: PTEN- Lipid phosphatase activity assay

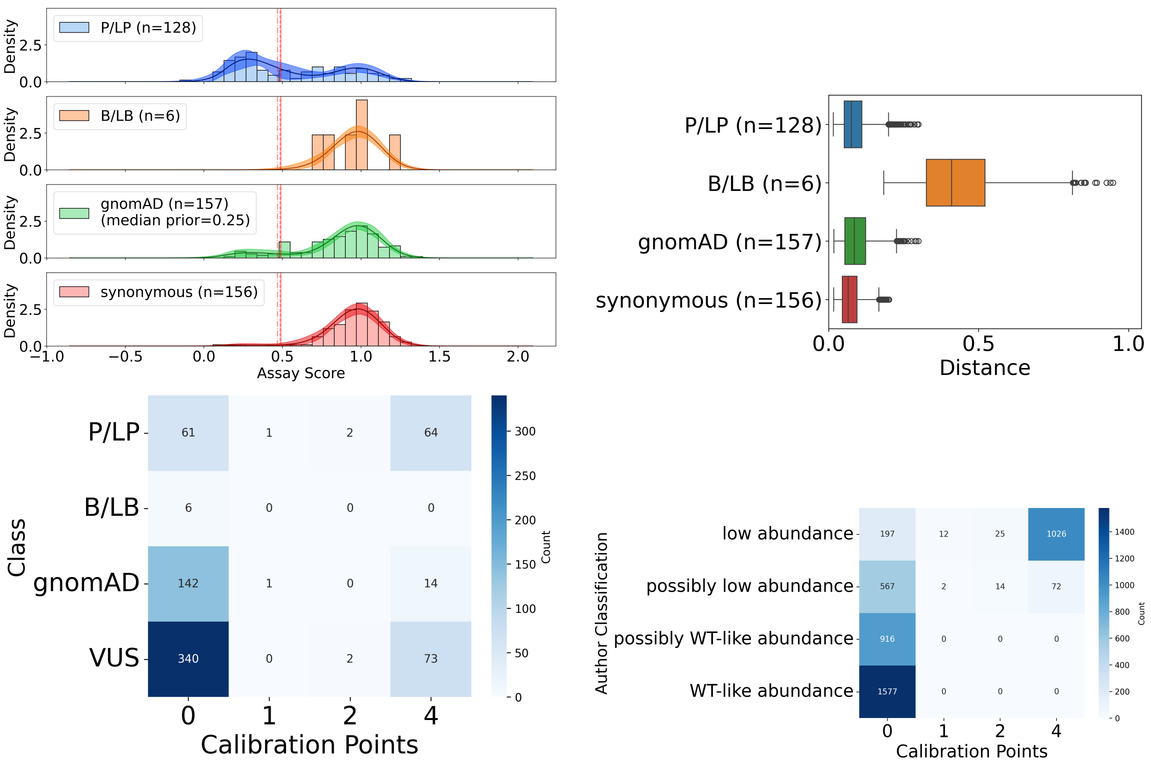

Figure S11: PTEN- VAMP-seq

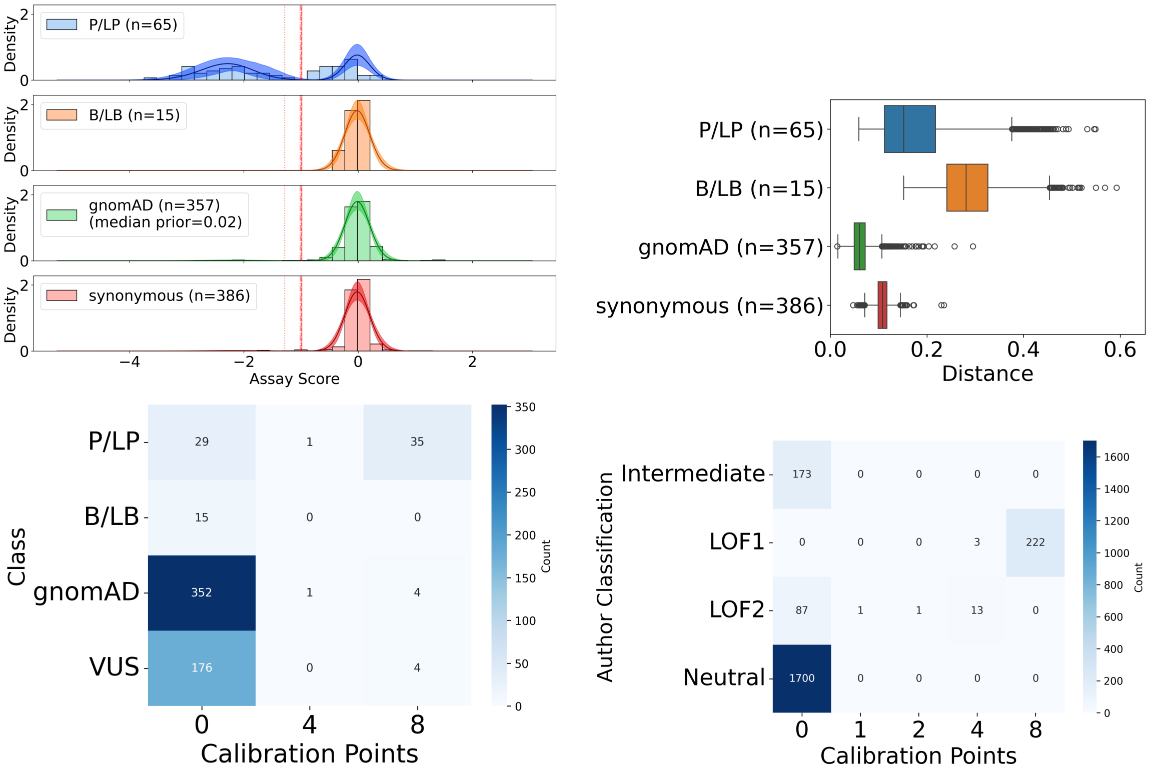

Figure S12: VHL- Saturation Genome Editing

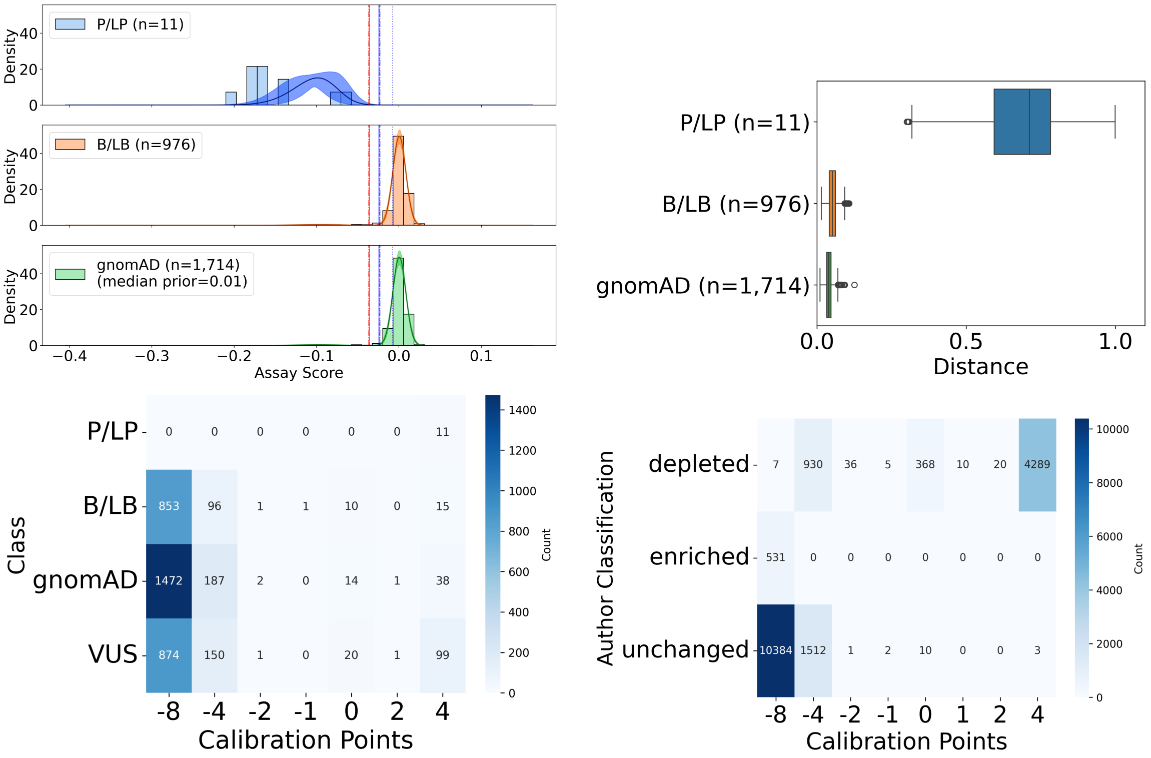

Figure S13: BAP1- Saturation Genome Editing

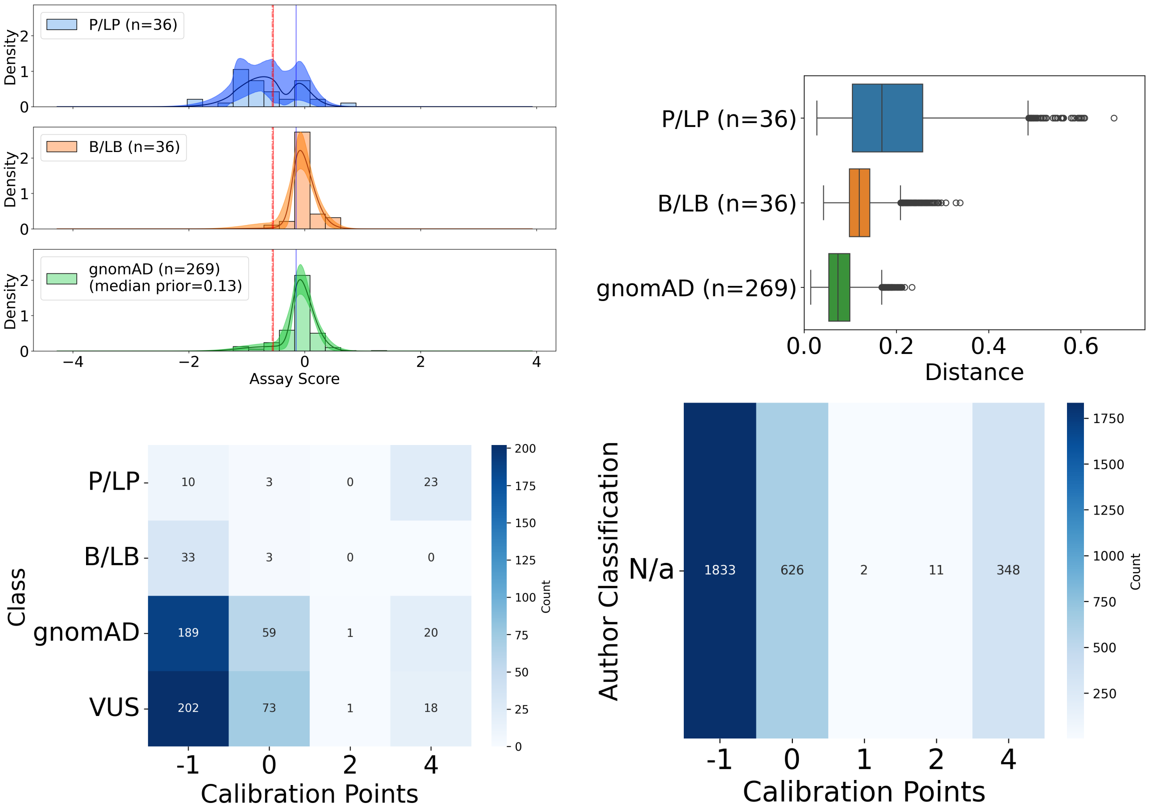

Figure S14: BRCA1- HDR

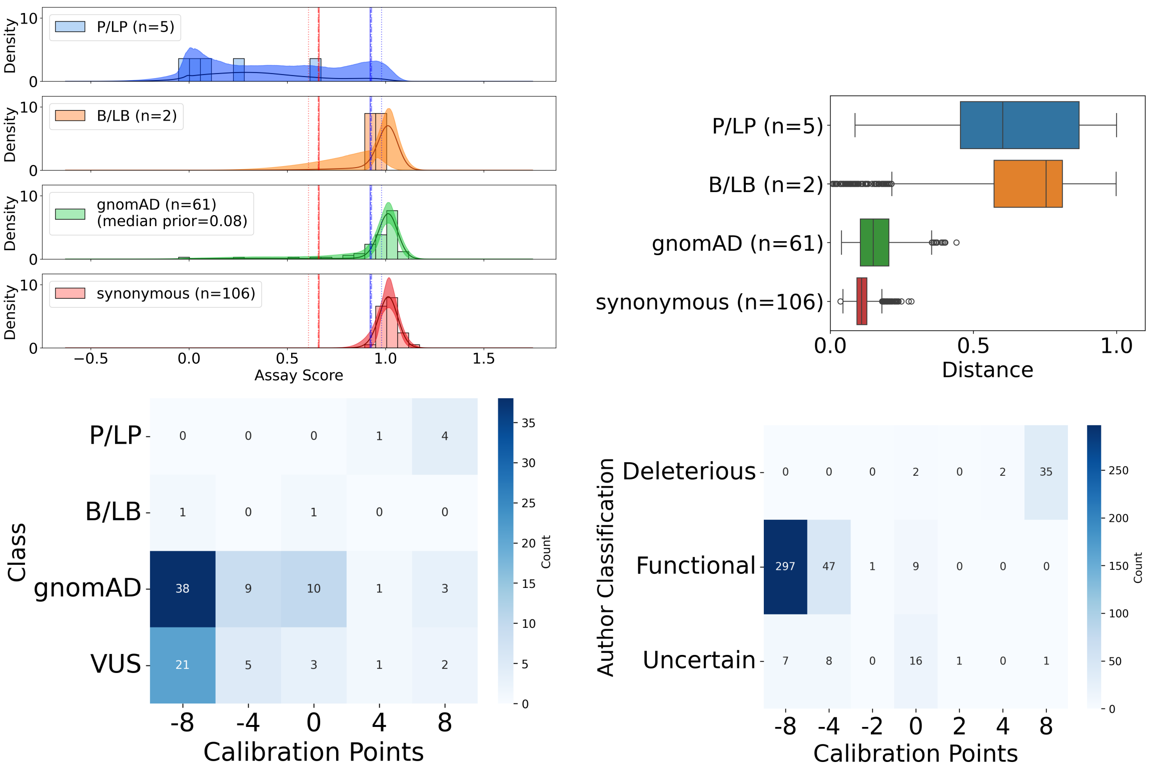

Figure S15: BRCA2- Saturation Prime Editing

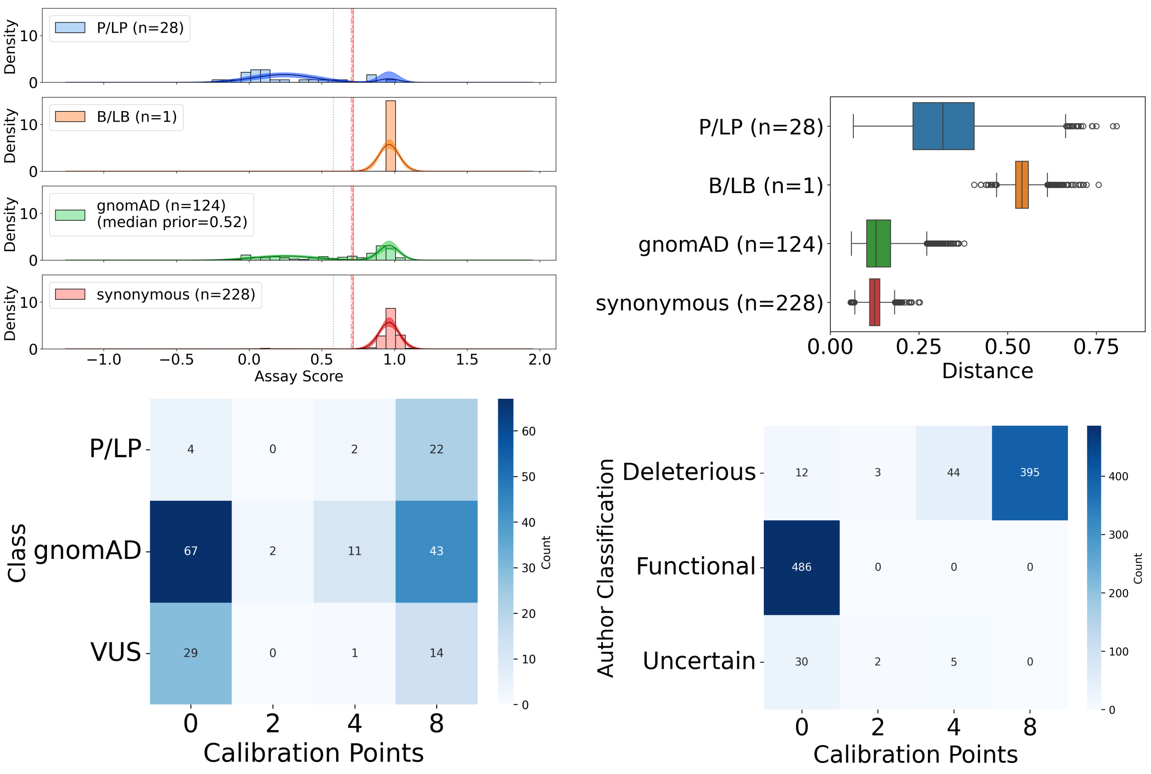

Figure S16: NPC1- Saturation Prime Editing (HEK293T)

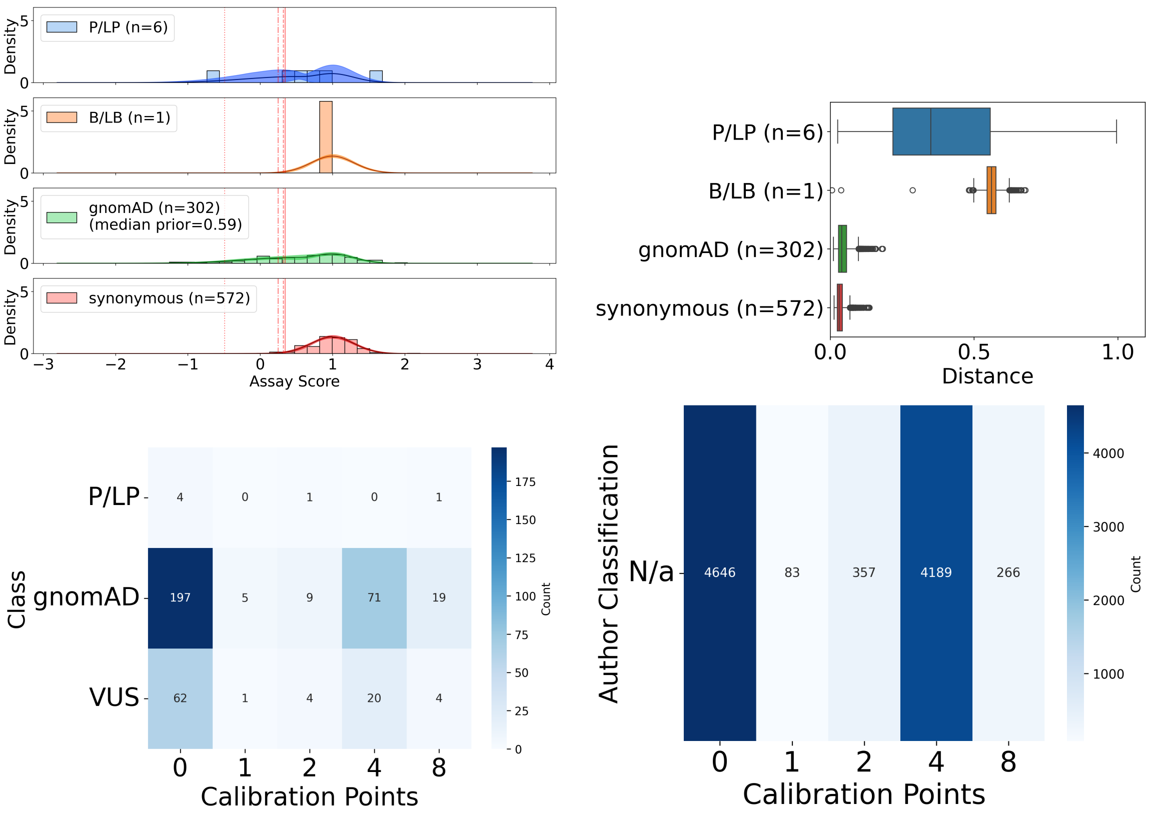

Figure S17: TPK1- Yeast complementation

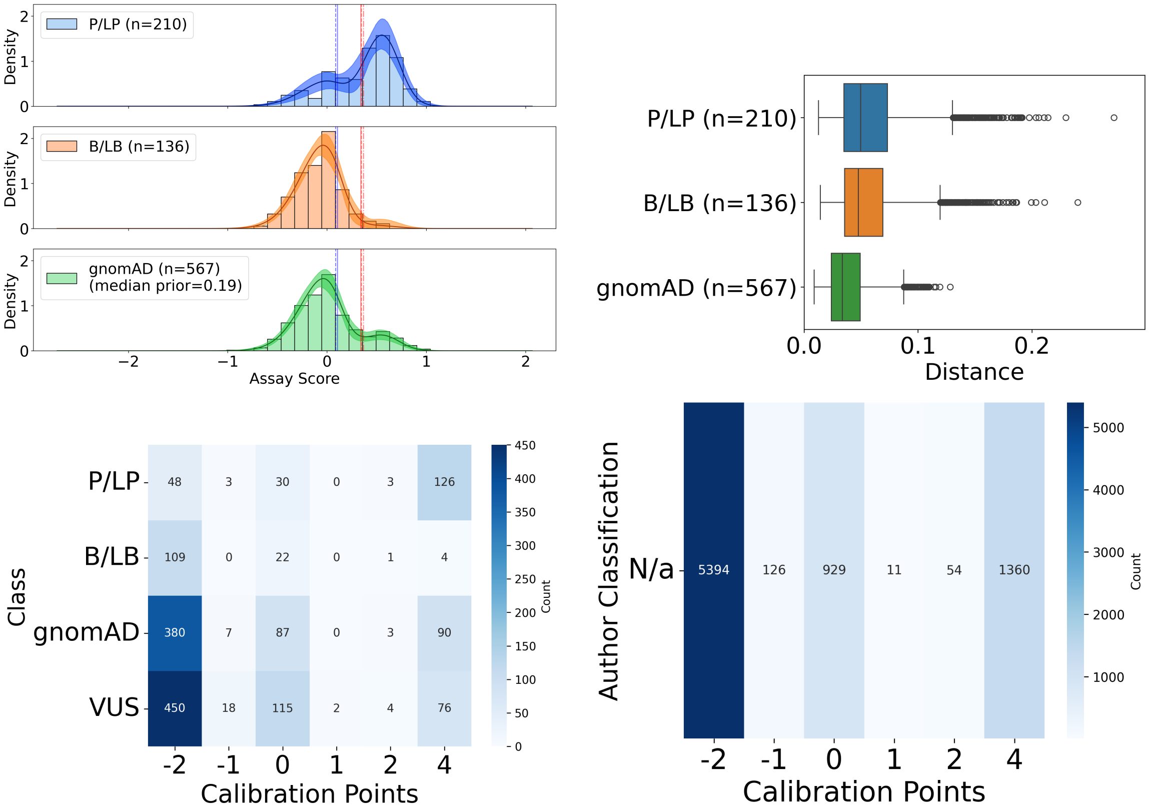

Figure S18: TP53- HDR Assay

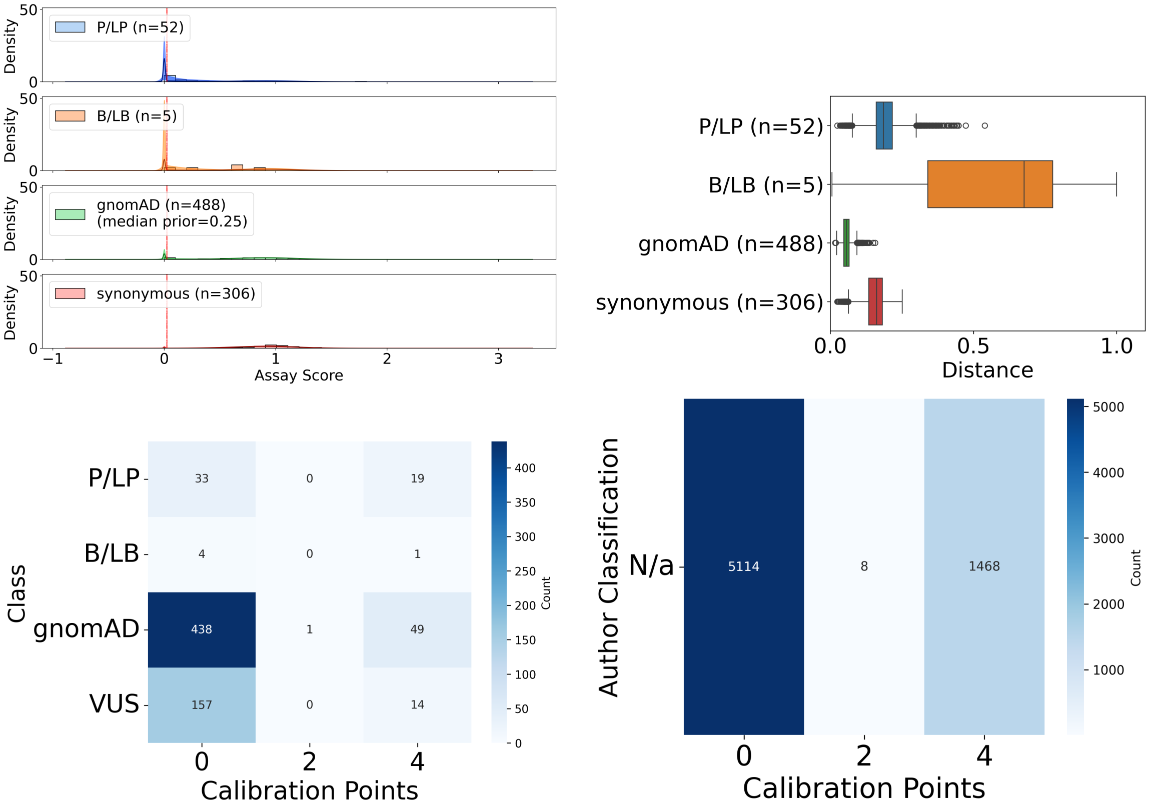

Figure S19: HMBS- Yeast complementation

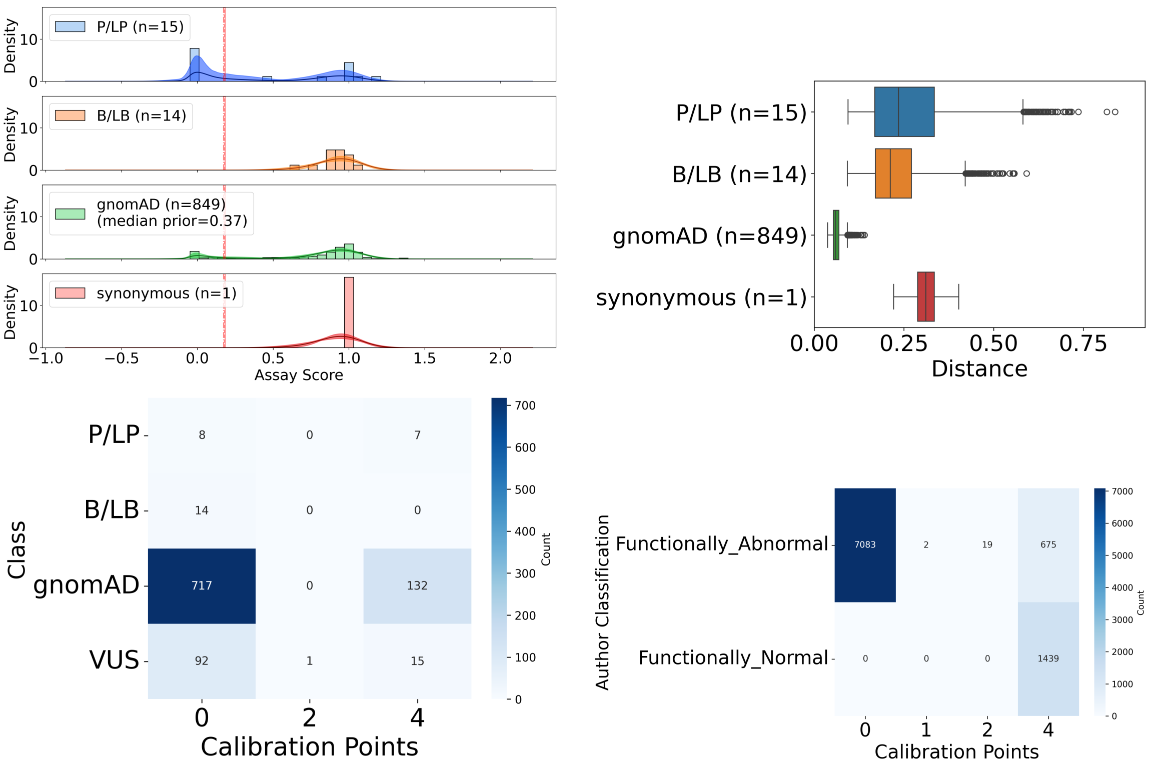

Figure S20: PRKN- VAMP-seq

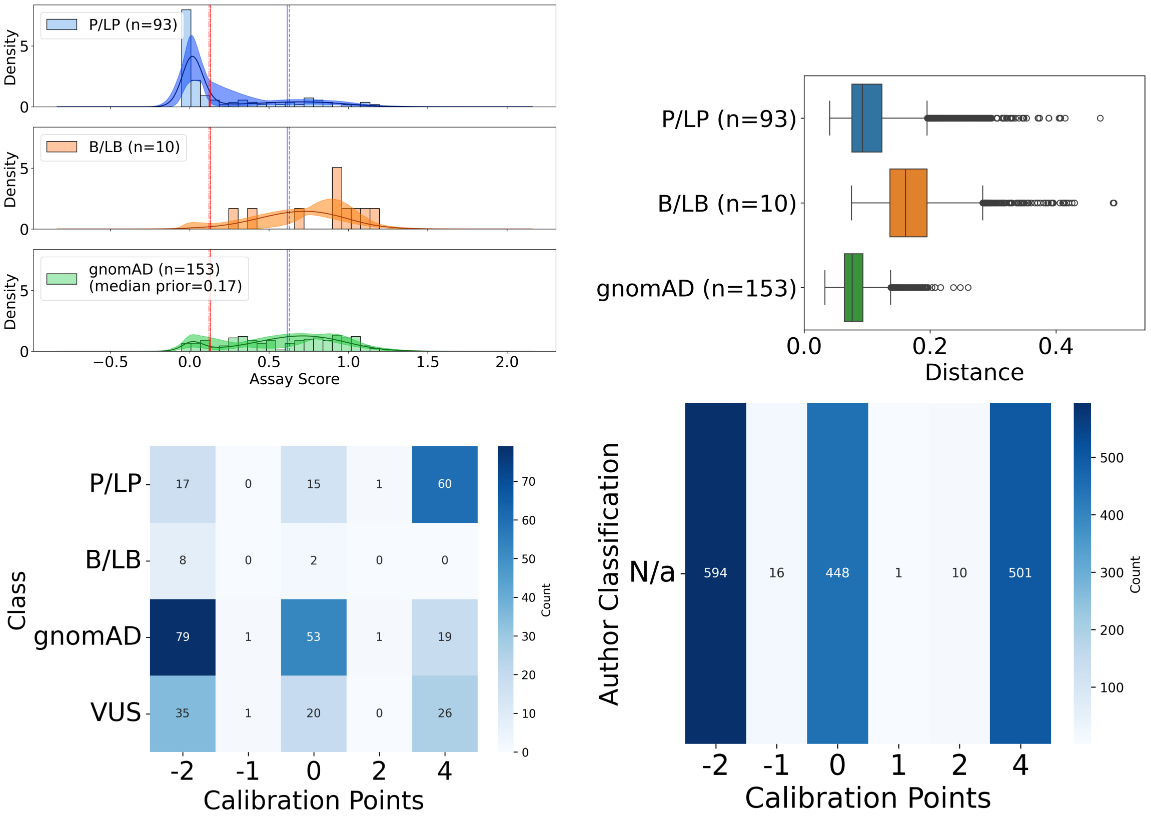

Figure S21: OTC- Yeast growth assay

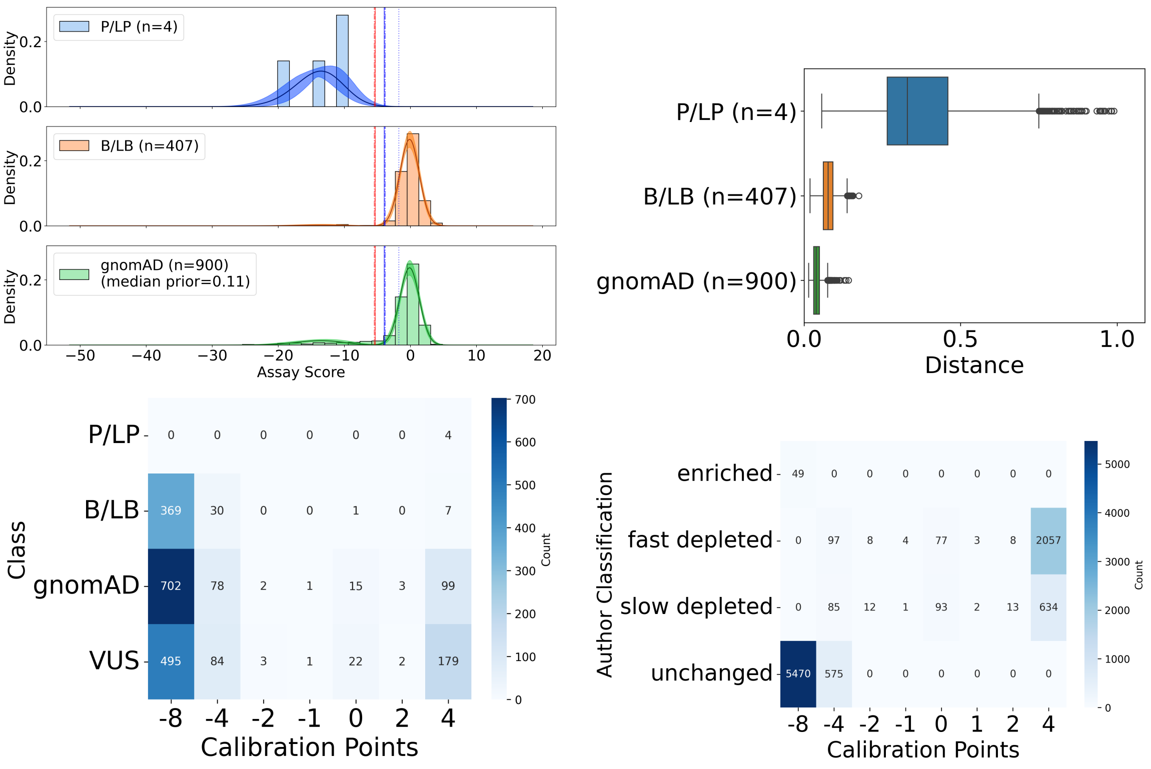

Figure S22: RAD51C- Saturation genome editing

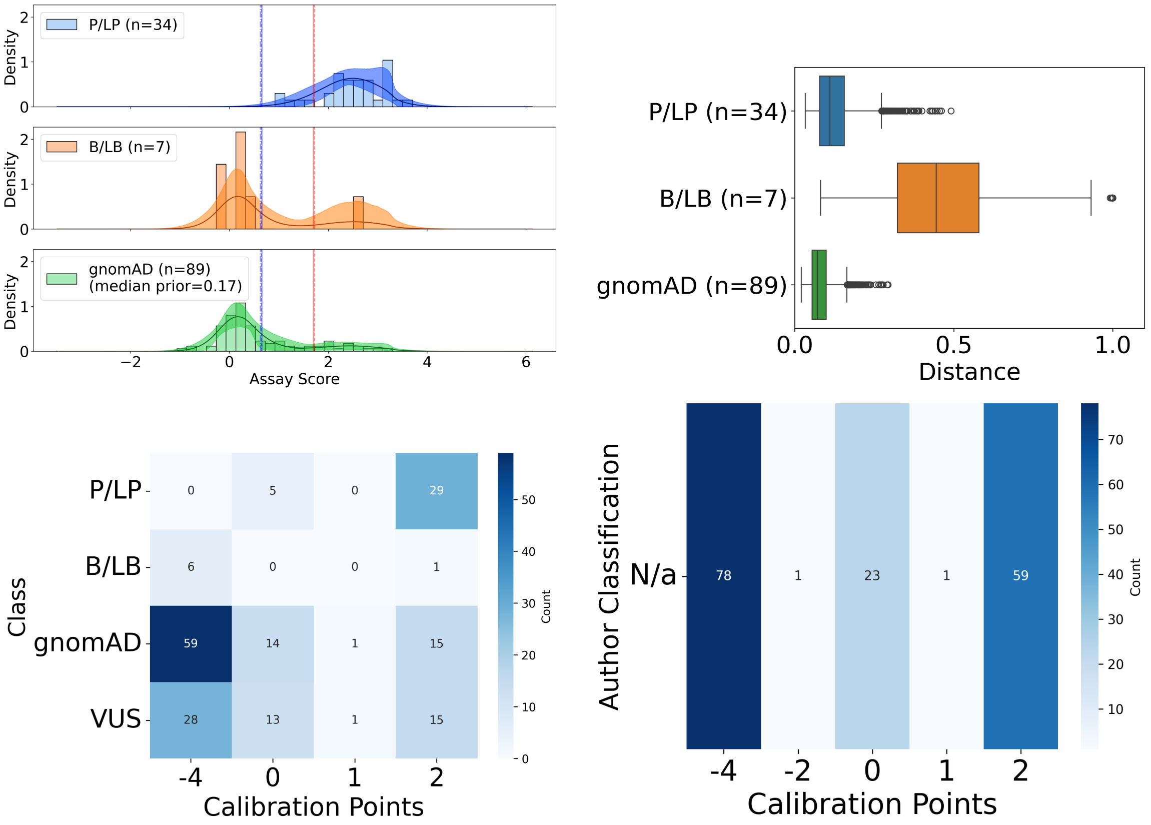

Figure S23: MSH2- Mutation rate assessment

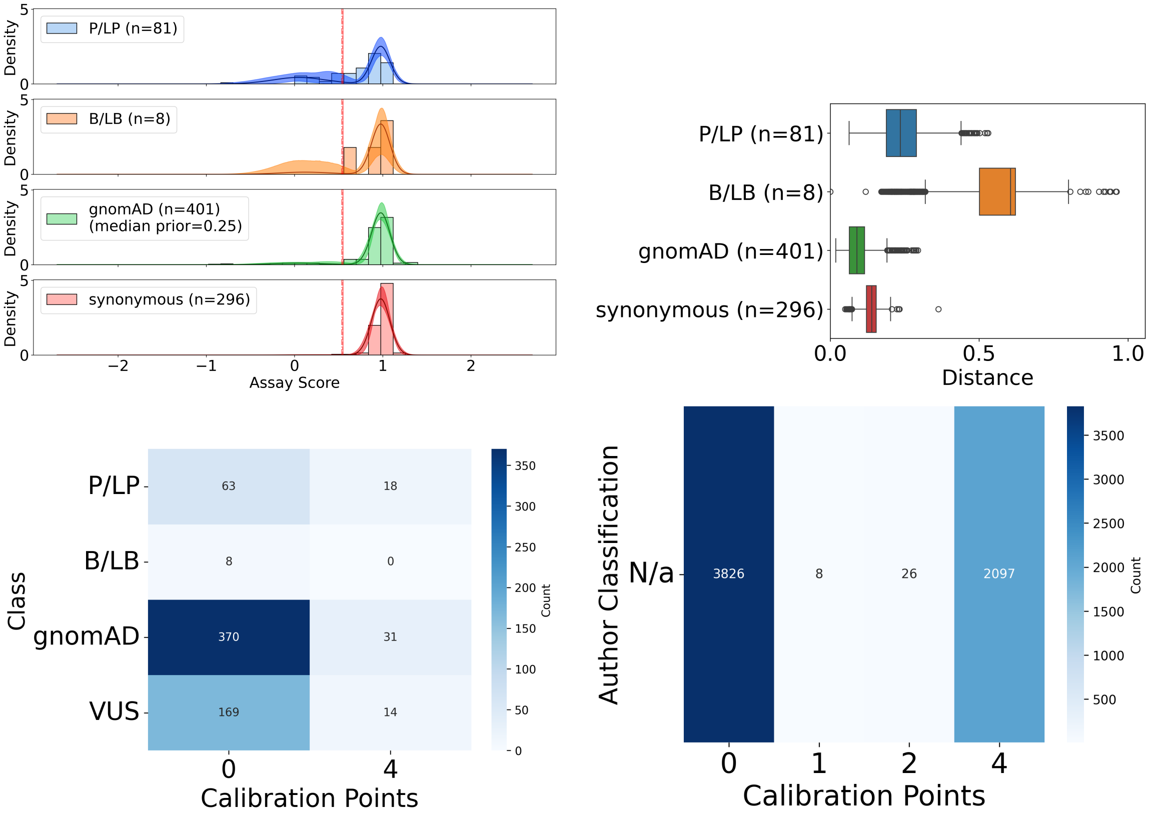

Figure S24: CALM1- Yeast complementation
